# Multi-omics reveals microbiota, metabolite, and immunological heterogeneity of age-related endotypes in type 1 diabetes

**DOI:** 10.1038/s41392-026-02724-2

**Published:** 2026-06-15

**Authors:** Lanxin Pan, Huiling Tan, Tong Yue, Yu Ding, Zhaohe Gu, Xulin Wang, Jing Wang, Tian Wei, Xiaoya Zhang, Yu Shi, Shiru Chang, Chuang Guo, Xueying Zheng, Jianping Weng

**Affiliations:** 1 Department of Endocrinology and Metabolism, The First Affiliated Hospital of USTC, Division of Life Sciences and Medicine, University of Science and Technology of China, Hefei, Anhui 230001 China; 2 Department of Rheumatology and Immunology, Institute of Endocrine and Metabolic Diseases, The First Affiliated Hospital of USTC, Division of Life Sciences and Medicine, University of Science and Technology of China, Hefei, Anhui 230001 China; 3 Anhui Provincial Key Laboratory of Metabolic Health and Panvascular Diseases, The First Affiliated Hospital of USTC, Division of Life Sciences and Medicine, University of Science and Technology of China, Hefei, 230001 China

**Keywords:** Immunological disorders, Metabolic disorders, Genome informatics, Predictive markers, Adaptive immunity

## Abstract

Type 1 diabetes (T1D) exhibits age-related heterogeneity in clinical progression and immune pathology, yet the underlying molecular mechanisms remain poorly understood. Here, we integrate microbiome, metabolome, lipidome, and transcriptome profiling from 108 newly diagnosed pediatric patients with T1D, along with 56 healthy controls, to investigate age-related endotypes. Patients were stratified into early-onset (E-T1D, <7 years), intermediate-onset (I-T1D, 7-12 years), and late-onset (L-T1D, ≥13 years) groups. Multi-omics analyses revealed distinct molecular signatures among T1D subgroups. The most enriched microbial signatures were the genus *Acetatifactor* in E-T1D, the phylum *Firmicutes A* in I-T1D, and the family *Bacteroidaceae* in L-T1D (Linear Discriminant Analysis scores = 3.49, 5.56, and 5.78, respectively). For metabolites, pipecolic acid increased most in E-T1D, testosterone in I-T1D, while N-acetylhomocitrulline was most enriched in L-T1D. Lipidomic profiling revealed subgroup-specific alterations, with increased levels of LPA(16:1) in E-T1D, TG(16:0/18:2/18:3) in I-T1D, and TG(18:0/18:1/18:1) in L-T1D. The proportion of peripheral B cells to total lymphocytes was the highest in E-T1D (median = 11.64%) and associated with upregulated immune-related pathways, lowest in L-T1D (median = 5.99%) and linked to metabolic processes, while I-T1D (median = 8.47%) exhibited intermediate features of both groups. Integration of multi-omics interaction networks and experimental validation revealed that the microbial species *Dialister invisus* may promote peripheral B cell proliferation via docosapentaenoic acid, potentially contributing to early-onset T1D. Together, these findings provide a molecular framework for understanding age-related T1D endotypes and suggest potential targets for precision intervention.

**Figure:**
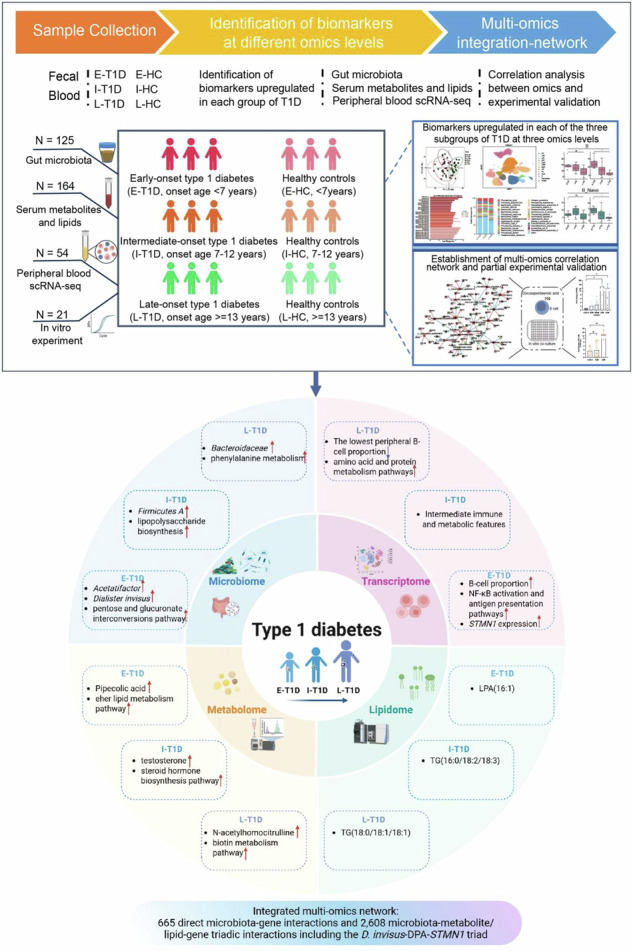
**Workflow and key findings of the study**.A multi-omics integration strategy was applied to newly diagnosed pediatric type 1 diabetes (T1D) patients stratified by age at diagnosis: early-onset (E-T1D), intermediate-onset (I-T1D), and late-onset (L-T1D), to delineate age-related T1D endotypes. Comprehensive profiling included gut microbiome, serum metabolome, lipidome, and peripheral immune transcriptome analyses. An integrated multi-omics interaction network revealed 665 direct microbiota–gene connections and 2,608 microbiota-metabolite/lipid-gene triadic interactions, highlighting a *D. invisus*-docosapentaenoic acid (DPA)-*STMN1* axis mediating B-cell activation in early-onset T1D

**Workflow and key findings of the study**.A multi-omics integration strategy was applied to newly diagnosed pediatric type 1 diabetes (T1D) patients stratified by age at diagnosis: early-onset (E-T1D), intermediate-onset (I-T1D), and late-onset (L-T1D), to delineate age-related T1D endotypes. Comprehensive profiling included gut microbiome, serum metabolome, lipidome, and peripheral immune transcriptome analyses. An integrated multi-omics interaction network revealed 665 direct microbiota–gene connections and 2,608 microbiota-metabolite/lipid-gene triadic interactions, highlighting a *D. invisus*-docosapentaenoic acid (DPA)-*STMN1* axis mediating B-cell activation in early-onset T1D

## Introduction

Type 1 diabetes (T1D) is an autoimmune disease characterized by hyperglycaemia resulting from insulin deficiency due to pancreatic β-cell destruction, and its development is influenced by both genetic susceptibility and environmental factors.^1,2^ T1D is one of the most common endocrine and metabolic diseases in childhood, with incidence peaking at 10–14 years of age, and a substantial burden of long-term complications and reduced life expectancy.^3–5^ Increasing evidence indicates that T1D is heterogeneous with respect to clinical features, rate of progression, residual β-cell function, immunopathology, and response to therapy.^6,7^ Among the factors associated with this heterogeneity, age at diagnosis has attracted particular attention.^8^ Histopathological studies^8–10^ have shown that children diagnosed before 7 years of age often display more aggressive insulitis, with higher levels of CD8^+^ T and CD20^+^ B cell infiltration, more extensive β cell loss, and abnormal proinsulin processing, whereas those diagnosed at 13 years of age or later tend to show the opposite pattern, and children diagnosed between 7 and 12 years of age may exhibit features of either phenotype. These histopathological observations are supported by findings from the Finnish Pediatric Diabetes Register.^11^ In this cohort, children diagnosed before 7 years of age had a higher prevalence of affected first-degree relatives, stronger HLA-conferred disease susceptibility, and a higher frequency of insulin autoantibodies. By contrast, those diagnosed at ≥13 years more often presented with a higher frequency of glutamic acid decarboxylase autoantibodies, longer symptom duration before diagnosis, and severe metabolic decompensation. Inshaw et al. also reported that a subset of T1D-associated variants predisposed more strongly to disease diagnosed under 7 years of age and that these variants were located near candidate genes with functions in both the immune system and pancreatic β-cells.^12^ Moreover, T1D diagnosed at a younger age has been associated with increased mortality and cardiovascular events, with the greatest excess risks seen in those diagnosed before 10 years of age.^13^ Together, these findings support the existence of age-related endotypes in T1D and emphasize the importance of accounting for heterogeneity associated with age at diagnosis.

The importance of studying age-related endotypes in T1D extends beyond disease classification. Heterogeneity in T1D has direct implications for disease prediction, prevention, and treatment, and is increasingly considered a key obstacle to precision medicine in this field.^14^ Patients differ substantially in their metabolic decline, preservation of endogenous insulin secretion, immune activation, and treatment response, suggesting that the current broad diagnostic category does not fully capture the underlying biology of the disease.^15,16^ The endotype concept has therefore gained increasing attention as a means of identifying subgroups of T1D that have distinct pathophysiological mechanisms and may be amenable to different interventions.^6^ This issue is especially relevant in T1D diagnosed during childhood and adolescence, where younger patients often show a more aggressive disease course and more rapid loss of β-cell function.^8–10^ A better understanding of age-related heterogeneity may help explain why disease progression varies so markedly among patients diagnosed at different ages, and may also inform the design of prevention strategies, biomarker-based risk stratification, and therapeutic trials.

Despite these advances, current knowledge of age-related T1D endotypes remains limited. Most previous work^8–12^ has focused on genetic susceptibility, autoantibody profiles, clinical characteristics at diagnosis, and pancreatic histopathology. These studies have been essential in establishing the concept of age-related endotypes, but they provide only a partial view of disease heterogeneity. T1D develops through interactions among genetic predisposition, immune dysregulation, β-cell stress, and environmental influences,^17^ and growing evidence suggests that the gut microbiota, circulating metabolites, lipid metabolism, and immune-cell functional states are all involved in this process.^18–22^ Variations in the microbiome have been increasingly reported in T1D, and metabolic and lipid disturbances, as well as immune-cell transcriptional alterations, are also linked to disease symptoms and progression. However, these molecular features have rarely been examined together in relation to age at diagnosis. In particular, it remains unclear whether children and adolescents diagnosed at different ages exhibit coordinated alterations across multiple molecular layers, or whether such alterations are connected through shared biological pathways. As a result, the molecular basis of age-related T1D endotypes remains insufficiently understood, and integrated multi-omics analysis is needed to provide a systems-level understanding of the mechanisms underlying these subgroups.

In the present study, we performed an integrated multi-omics analysis of pediatric patients with newly diagnosed T1D stratified into early-onset (<7 years), intermediate-onset (7-12 years), and late-onset (≥13 years) groups according to age at diagnosis. We integrated gut microbiome, serum metabolomic, serum lipidomic, and peripheral immune transcriptomic profiles to define molecular features associated with age-related T1D endotypes. Rather than describing differences within individual omics datasets alone, we examined potential links among molecular layers using multi-omics network analysis and experimental validation. Our study provides an integrated view of age-related heterogeneity in pediatric T1D and identifies distinct molecular and immune features across age-defined subgroups. These findings extend the current framework of age-related T1D endotypes and provide a basis for future biomarker development and mechanism-informed precision intervention.

## Results

### Clinical characteristics of the study sample

To systematically investigate molecular heterogeneity associated with age at diagnosis in pediatric T1D, we collected blood, fecal, and peripheral blood mononuclear cells (PBMCs) from 108 newly diagnosed T1D patients and 56 age- and sex-matched healthy controls, and performed integrated analyses of the gut microbiome, serum metabolome, serum lipidome, and single-cell PBMC transcriptome. We first explored the associations between age at diagnosis and clinical parameters (Supplementary Tables 1–3). The frequency of high-risk HLA genotypes was elevated in the T1D groups compared to healthy controls (HC), with a decreasing trend across the E-T1D, I-T1D, and L-T1D subgroups (*P* = 0.015). Antibody positivity and ketosis at diagnosis did not differ significantly among T1D subgroups. Fasting C-peptide levels showed an age-related increase (*P* < 0.001), while lipid profiles indicated more pronounced metabolic abnormalities in L-T1D (*P* = 0.005). No statistical differences in high-density lipoprotein cholesterol and total cholesterol were observed in the HC groups. Thyroid peroxidase antibody levels were significantly higher in T1D compared to HC, with a trend toward increased levels in the L-T1D subgroup.

Together, these findings highlight age-related heterogeneity in HLA risk, β-cell function, and metabolic profiles among pediatric T1D individuals.

### The microbial signatures in age-related endotypes of T1D

**Fig. 1:**
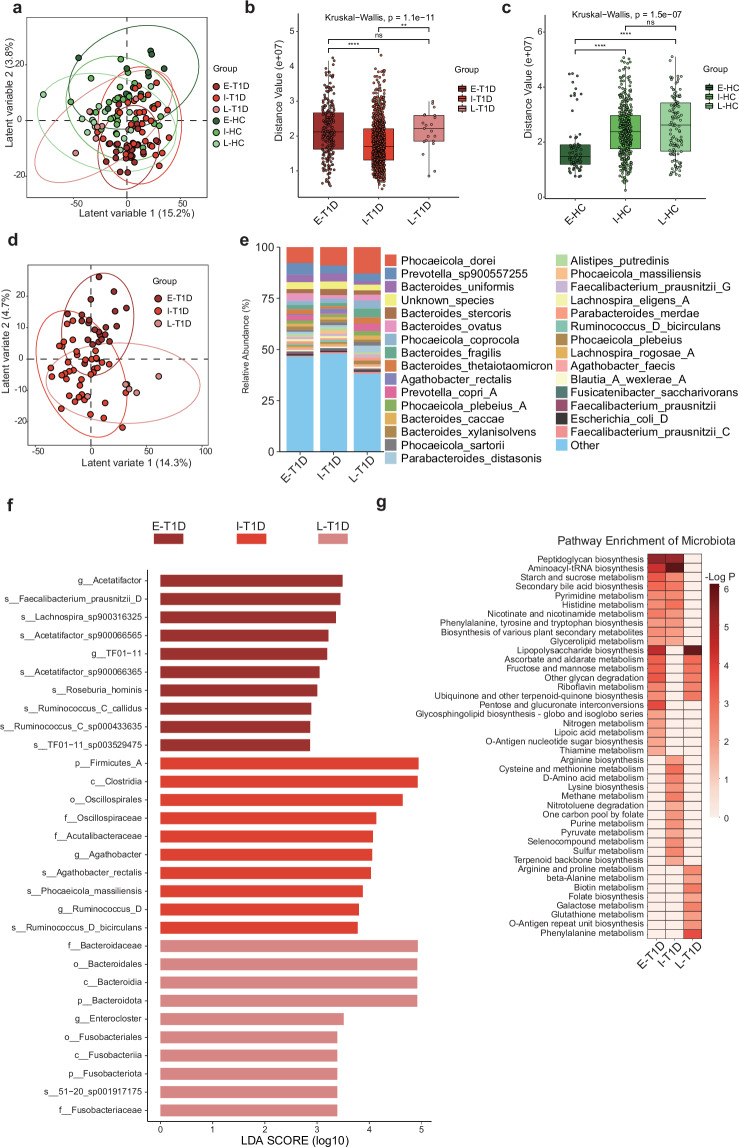
The microbial signatures in age-related endotypes of T1D. **a** PLS-DA plot showing gut microbiota distribution across six groups: E-T1D, I-T1D, L-T1D, and age-matched healthy controls (E-HC, I-HC, L-HC). **b** β-diversity comparison among E-T1D, I-T1D, and L-T1D subgroups based on Euclidean distance. **c** β-diversity comparison among E-HC, I-HC, and L-HC subgroups based on Euclidean distance. **d** PLS-DA plot showing gut microbiota distribution across E-T1D, I-T1D, and L-T1D subgroups. **e** Relative abundance of dominant microbial species across E-T1D, I-T1D, and L-T1D subgroups. **f** LDA scores of differentially enriched taxa among the E-T1D, I-T1D, and L-T1D subgroups, identified by LEfSe after subtracting taxa enriched in the corresponding HC subgroups (E-HC, I-HC, and L-HC) to remove age-related effects. **g** Pathway enrichment analysis of subgroup-specific microbial features among E-T1D, I-T1D, and L-T1D groups. Statistical comparisons were performed using the Kruskal–Wallis test. Asterisks indicate significance levels: *P* < 0.05 (*), *P* < 0.01 (**), *P* < 0.001 (***), and *P* < 0.0001 (****); “ns” indicates not significant. In panels **a**–**d** and **f**–**g**, colors represent different T1D or HC subgroups. In panel **e**, colors represent different microbial species. In panel **h**, color corresponds to the -log(*P*) value

The partial least squares discriminant analysis (PLS-DA) showed that gut microbiota profiles differed among the E-T1D, I-T1D, L-T1D, E-HC, I-HC, and L-HC subgroups (Fig. 1a). The β-diversity among the E-T1D, I-T1D, and L-T1D subgroups, as well as among the E-HC, I-HC, and L-HC subgroups was significantly different and shown in Fig. 1b, c. The distribution of microbiota profiles among the three subgroups of T1D and HC is displayed in Fig. 1d and Supplementary Fig. 1a, which clearly showed separation among subgroups. Interestingly, I-T1D appeared as a transitional state, partially overlapping with both E-T1D and L-T1D. Compositional differences among T1D subgroups were further illustrated by relative abundance analysis (Fig. 1e).

Differential microbiota between the E-T1D, I-T1D, and L-T1D subgroups were identified by Linear discriminant analysis Effect Size (Lefse) (Supplementary Fig. 1b). To eliminate the potential confounding effect of age on microbiota characteristics across T1D subgroups, differential microbiota among the E-HC, I-HC, and L-HC subgroups were also identified (Supplementary Fig. 1c). Subsequently, the differential microbiota of each T1D subgroup were subtracted by the differential microbiota of the corresponding HC subgroup to identify specific microbial signatures of each T1D subgroup (Fig. 1f). Finally, the Lefse results showed that the genus *Acetatifactor*, the phylum *Firmicutes A*, and the family *Bacteroidaceae* were the most enriched microbial signatures in the E-T1D, I-T1D, and L-T1D subgroups, respectively (LDA = 3.49, 5.56, and 5.78). We also identified specific microbial signatures across T1D subgroups at different taxonomic levels (Supplementary Fig. 2a–f). Functional KEGG pathway enrichment analysis was performed on the specific fecal microbiomes of three T1D subgroups. The pentose and glucuronate interconversions and phenylalanine metabolism pathways had the highest reporter scores in the E-T1D and L-T1D subgroups, respectively (reporter score = 3.71 and 3.65) (Fig. 1g).

We next compared gut microbiota composition (Supplementary Fig. 3a–c) and β-diversity (Supplementary Fig. 3g–i) between each T1D subgroup and its age-matched HC subgroup, and observed significant differences across all comparisons. Differential taxa were identified (Supplementary Fig. 3d–f), and functional enrichment analysis was conducted (Supplementary Fig. 4a–c). In the E-T1D subgroup, the genus *Coprococcus* was most prominently enriched compared to E-HC (LDA = 3.39), accompanied by enrichment of the galactose metabolism pathway. In I-T1D, the phylum *Firmicutes A* showed the strongest enrichment (LDA = 4.67), with functional upregulation of lipopolysaccharide biosynthesis. In L-T1D, the species *Bacteroides fragilis* was markedly increased compared to L-HC (LDA = 4.29), with aminoacyl-tRNA biosynthesis identified as the most enriched pathway.

Overall, these data indicate that age-related T1D endotypes are associated with distinct gut microbial compositions and functional profiles.

### The metabolic signatures in age-related endotypes of T1D

**Fig. 2:**
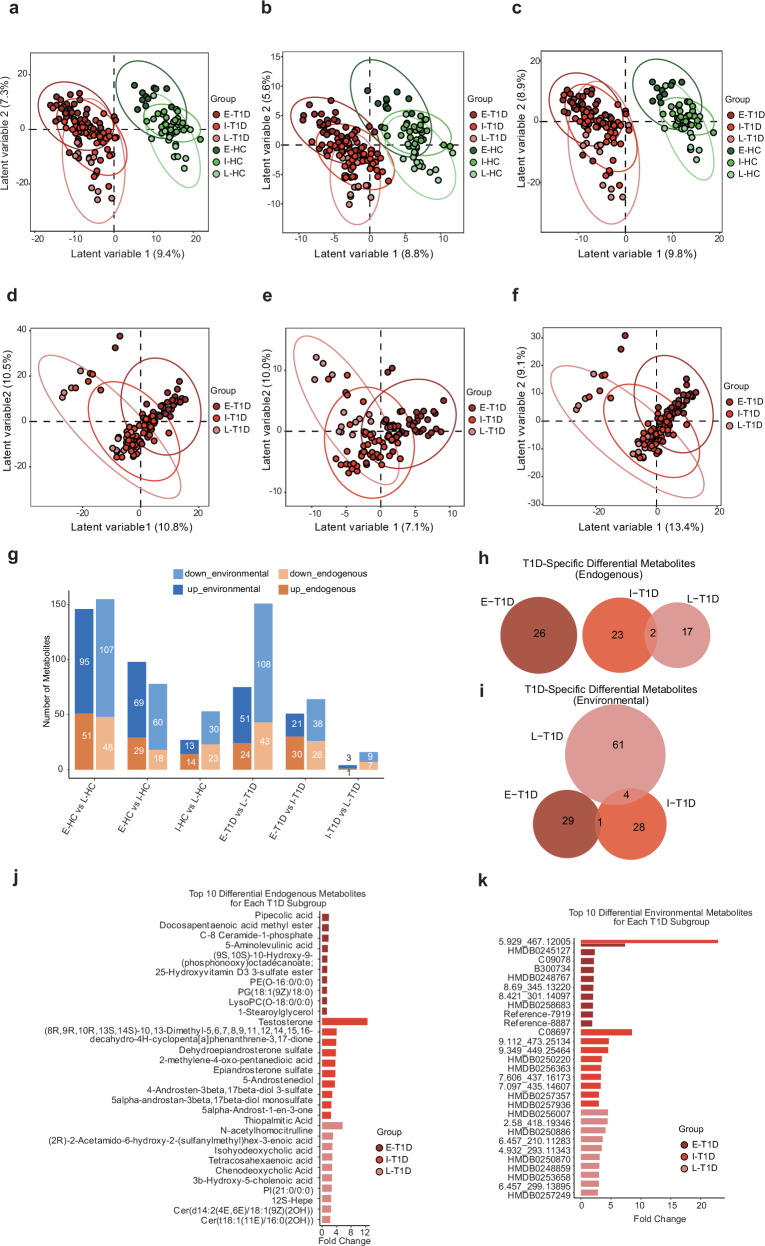
The metabolic signatures in age-related endotypes of T1D. PLS-DA plots of serum metabolite distributions among six groups (E-T1D, I-T1D, L-T1D, E-HC, I-HC, L-HC) for (**a**) all identified metabolites, (**b**) endogenous metabolites, and (**c**) environmental metabolites. PLS-DA plot showing serum metabolite distribution across E-T1D, I-T1D, and L-T1D subgroups for (**d**) all identified metabolites, (**e**) endogenous metabolites, and (**f**) environmental metabolites. **g** Number of significantly altered metabolites in each pairwise group comparison among T1D and HC groups, categorized by endogenous and environmental sources. Venn diagram showing overlap of T1D-specific differentially upregulated metabolites among E-T1D, I-T1D, and L-T1D, categorized by endogenous (**h**) and environmental (**i**) sources. Top 10 specifically upregulated metabolites for each T1D subgroup based on fold change categorized by endogenous (**j**) and environmental (**k**) sources. Statistical significance for metabolite selection was defined as fold change ≥ 1.2 or ≤ 0.83 and q-value < 0.05. In panels **a**, **c**, and **d**, colors represent different T1D or HC subgroups. In panel **b**, colors indicate direction of regulation. In panel **e**, colors correspond to -log(*P*) value

Using non-targeted liquid chromatography-tandem mass spectrometry, we identified 3882 plasma metabolites, which we manually annotated as 1096 endogenous and 2786 environmental metabolites. The results of the PLS-DA analysis indicated distinct serum metabolite profiles among the E-T1D, I-T1D, L-T1D, E-HC, I-HC, and L-HC subgroups, whether across all metabolites, endogenous metabolites, or environmental metabolites. I-T1D was positioned between E-T1D and L-T1D, partially overlapping with both, suggesting a potential intermediate metabolic phenotype (Fig. 2a–f, Supplementary Fig. 5a–c).

Differential metabolites were selected based on the VIP values from the Orthogonal Projections to Latent Structures Discriminant Analysis (OPLS-DA), as well as the fold change (FC) and q-value from univariate analysis (VIP ≥ 1, FC ≥ 1.2 or ≤ 0.83, and q-value < 0.05). The numbers of significantly increased or decreased metabolites between subgroups are shown in Fig. 2g, categorized by endogenous and environmental sources. To eliminate the potential confounding effect of age on metabolic profiles across T1D subgroups, differential metabolites were also identified among the E-HC, I-HC, and L-HC subgroups. For each subgroup, metabolites that showed significantly higher levels in any pairwise comparison with the other two subgroups were defined as characteristic metabolites of that subgroup, and results from the corresponding age-matched HC subgroup were subtracted to obtain subgroup-specific metabolic signatures. Subgroup-specific metabolites were identified in the E-T1D, I-T1D, and L-T1D subgroups, comprising 26, 25, and 19 endogenous metabolites, respectively, alongside 30, 33, and 65 environmental metabolites (Fig. 2h, i). Among endogenous metabolites, pipecolic acid showed the greatest increase in E-T1D (FC = 1.87, q-value = 3.72e-5, E-T1D vs. L-T1D), testosterone in I-T1D (FC = 12.51, q-value = 5.97e-8, I-T1D vs. E-T1D), and N-acetylhomocitrulline (FC = 5.63, q-value = 0.022, L-T1D vs. E-T1D) in the L-T1D subgroup (Fig. 2j). Among environmental metabolites, 5.929_467.12005 (N-[2-(4-methoxyphenoxy)-5-(trifluoromethyl)phenyl]-5-methyl-2-phenyl-1,3-oxazole-4-carboxamide) showed the most pronounced alteration (FC = 7.35, q-value = 0.026, E-T1D vs. L-T1D) in the E-T1D subgroup, which was also markedly elevated in the I-T1D group (FC = 22.87, q-value = 0.012, I-T1D vs. L-T1D). And in the L-T1D subgroup, HMDB0256007 (Hypromellosum) exhibited the greatest elevation (FC = 4.54, q-value = 0.019, L-T1D vs. E-T1D) (Fig. 2k). Additionally, through functional pathway enrichment analysis of endogenous metabolites, we found that ether lipid metabolism was the top enriched pathway within the E-T1D subgroup (*P* = 0.049), and biotin metabolism showed the highest enrichment within the L-T1D subgroup (*P* = 0.043) (Supplementary Fig. 5d). For KEGG functional pathways enriched in the metagenome across T1D subgroups, we examined the distribution of metabolites within each pathway across T1D groups in the metabolome. In the pentose and glucuronate interconversion pathway, which was upregulated in E-T1D, xylonic acid showed significant upregulation in E-T1D (Supplementary Fig. 6a). In the phenylalanine metabolism pathway, which was upregulated in L-T1D, phenylacetaldehyde, l-5-hydroxytryptophan, and 3-phenyllactic acid exhibited significant upregulation in L-T1D (Supplementary Fig. 6b–d).

We also performed subgroup comparisons and differential metabolite screening for E-T1D vs. E-HC, I-T1D vs. I-HC, and L-T1D vs. L-HC separately at endogenous and environmental levels (Supplementary Figs. 7a, b, and 8a–d). The results showed that in the E-T1D subgroup, 252 metabolites were upregulated and 268 metabolites were downregulated compared to the E-HC subgroup, with the most significantly enriched pathway being ABC transporters (Supplementary Fig. 7c, d). In the I-T1D subgroup, 445 metabolites were upregulated and 421 metabolites were downregulated compared to the I-HC subgroup, with the most significantly enriched pathway being bile secretion (Supplementary Fig. 7e, f). In the L-T1D subgroup, 175 metabolites were upregulated and 237 metabolites were downregulated compared to the L-HC subgroup, with the most significantly enriched pathway being tryptophan metabolism (Supplementary Fig. 7g, h).

Taken together, these findings indicate distinct metabolic alterations across age-related T1D endotypes at both the endogenous and environmental levels, with the intermediate-onset subgroup showing features shared with both early- and late-onset T1D.

### The lipidomic signatures in age-related endotypes of T1D

**Fig. 3:**
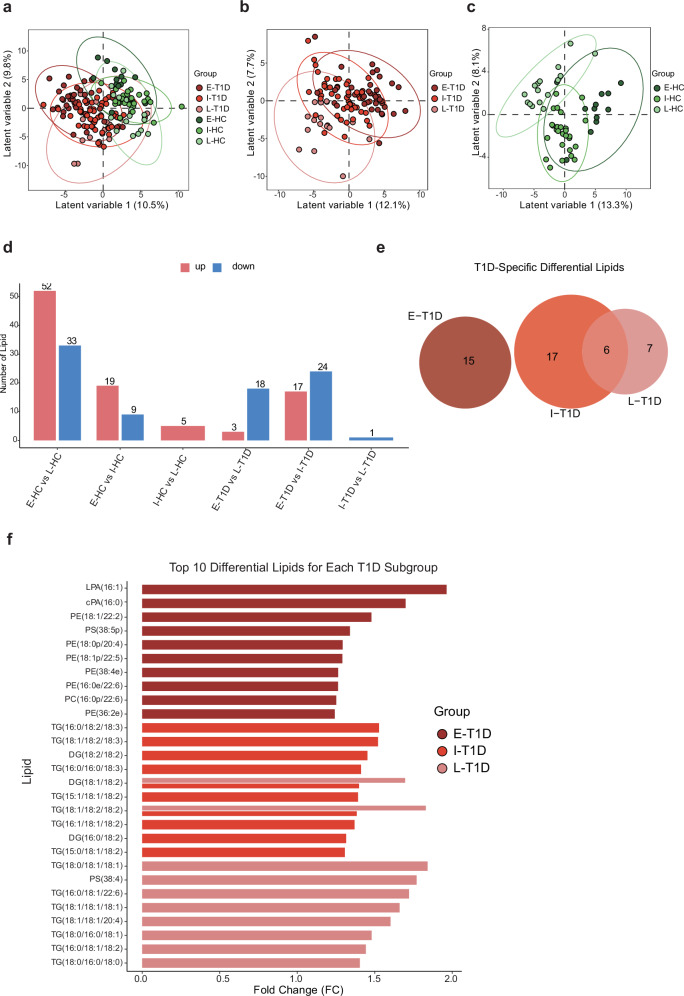
The lipidomic signatures in age-related endotypes of T1D. **a** PLS-DA plot showing serum lipid distribution across six groups: E-T1D, I-T1D, L-T1D, and age-matched healthy controls (E-HC, I-HC, L-HC). **b** PLS-DA plot showing serum lipid distribution across E-T1D, I-T1D, and L-T1D subgroups. **c** PLS-DA plot showing serum lipid distribution across E-HC, I-HC, and L-HC subgroups. **d** Number of significantly altered lipids in each pairwise group comparison among T1D and HC groups. **e** Venn diagram showing overlap of T1D-specific differentially upregulated lipids among E-T1D, I-T1D, and L-T1D. **f** Top 10 significantly upregulated lipids for each T1D subgroup based on fold change. Statistical significance for lipid selection was defined as fold change ≥ 1.2 or ≤ 0.83 and q-value < 0.05. In panels **a**–**c** and **e**–**f**, colors represent different T1D or HC subgroups. In panel **d**, colors indicate direction of regulation

Serum lipid profiles also differed across the six subgroups, as shown by PLS-DA. In line with the metabolomic data, the I-T1D group appeared intermediate between E-T1D and L-T1D in its lipidomic pattern (Fig. 3a–c). The numbers of significantly increased or decreased lipids between subgroups are presented in Fig. 3d. To account for possible age-related influences on lipid composition, lipidomic differences were also examined among the E-HC, I-HC, and L-HC subgroups. Within each T1D subgroup, lipids that were significantly increased in comparison with either of the other two subgroups were regarded as representative lipids of that subgroup. Age-matched HC results were then used as a reference to remove shared alterations and define lipid signatures specific to each T1D subgroup. A total of 15 lipidomic signatures were identified in E-T1D, 23 in I-T1D, and 13 in L-T1D (Fig. 3e). The most significantly altered lipids in each group were LPA(16:1) for E-T1D (FC = 1.96, q-value = 0.023, E-T1D vs. L-T1D), TG(16:0/18:2/18:3) for I-T1D (FC = 1.53, q-value = 0.006, I-T1D vs. E-T1D), and TG(18:0/18:1/18:1) for L-T1D (FC = 1.84, q-value = 0.007, L-T1D vs. E-T1D) (Fig. 3f).

We also compared each T1D subgroup with its corresponding healthy control group to identify differential lipids (Supplementary Fig. 9a, b, g, and h). The results showed that in the E-T1D subgroup, 51 lipids were upregulated and 40 lipids were downregulated compared to the E-HC subgroup (Supplementary Fig. 9c). In the I-T1D subgroup, 84 lipids were upregulated and 63 lipids were downregulated compared to the I-HC subgroup, with the most significantly enriched pathway being the biosynthesis of unsaturated fatty acids pathway (Supplementary Fig. 9d, e). In the L-T1D subgroup, 2 lipids were upregulated and 1 lipid was downregulated compared to the L-HC subgroup (Supplementary Fig. 9f).

These results demonstrate that lipid dysregulation differs substantially among age-related T1D endotypes, highlighting distinct lipidomic alterations across subgroups.

### The single-cell RNA-seq signatures in age-related endotypes of T1D

**Fig. 4:**
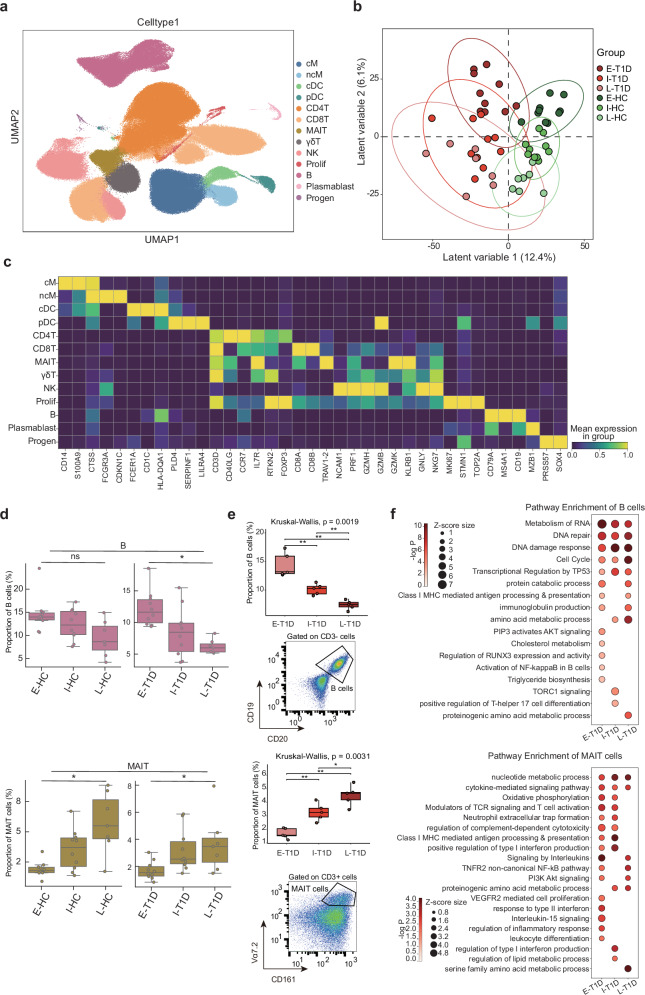
The single-cell RNA-seq signatures in age-related endotypes of T1D. **a** UMAP projection showing 13 immune cell types identified from single-cell RNA-seq of PBMCs, including CD14^+^ classical monocytes (cM), CD16^+^ non-classical monocytes (ncM), conventional dendritic cells (cDC), plasmacytoid dendritic cells (pDC), CD4^+^ T cells (CD4T), CD8^+^ T cells (CD8T), mucosal-associated invariant T cells (MAIT), Gamma Delta T cells (γδ T), natural killer cells (NK), proliferating T/NK cells (Prolif), B cells (B), plasmablasts (Plasmablast), and progenitor cells (Progen). Colors represent different cell types. **b** PLS-DA plot of immune transcriptomic profiles across six groups: E-T1D, I-T1D, L-T1D, and age-matched controls (E-HC, I-HC, L-HC). Colors represent different T1D or HC subgroups. **c** Heatmap of representative marker gene expression across the 13 immune cell types. Colors represent scaled average expression values per group. **d** Proportions of peripheral B and MAIT cells to total lymphocytes across T1D and HC subgroups based on single-cell RNA sequencing data. **e** Proportions of peripheral B and MAIT cells to total lymphocytes across T1D and HC subgroups measured by flow cytometry. **f** Pathway enrichment analysis of DEGs in B and MAIT cells from E-T1D, I-T1D, and L-T1D groups. Colors indicate -log(*P*) values, and dot size indicates Z-score. For single-cell RNA-seq data, differences in cell proportions were assessed using a weighted least square model to account for sample-level variability, and *P* values were adjusted using the Bonferroni method. For flow cytometry data, statistical comparisons were performed using the Kruskal-Wallis test followed by pairwise Wilcoxon tests with Holm correction for multiple testing. Asterisks indicate significance levels: *P* < 0.05 (*), *P* < 0.01 (**); “ns” indicates not significant

From samples with microbiome, metabolome, and lipidome data, we selected 10 E-T1D, 10 I-T1D, and 7 L-T1D individuals based on age at diagnosis, paired with 10 E-HC, 10 I-HC, and 7 L-HC healthy controls matched for age and sex (Supplementary Tables 4 and 5). A total of 54 samples were subjected to single-cell RNA sequencing of PBMCs, yielding transcriptional profiles of 32,177 genes in 376,996 cells (Supplementary Fig. 10a, b). We identified 13 immune cell types: CD14^+^ classical monocytes (cM), CD16^+^ non-classical monocytes (ncM), conventional dendritic cells (cDC), plasmacytoid dendritic cells (pDC), CD4^+^ T cells (CD4T), CD8^+^ T cells (CD8T), mucosal-associated invariant T cells (MAIT), Gamma Delta T cells (γδ T), natural killer cells (NK), proliferating T/NK cells (Prolif), B cells (B), plasmablasts (Plasmablast), and progenitor cells (Progen) (Figs. 4a and c, Supplementary Figs. 10c, d). The PLS-DA analysis was highly concordant with previously described omics data, revealing distinct between-group differences and within-group continuity among E-T1D, I-T1D, L-T1D, E-HC, I-HC, and L-HC (Fig. 4b, Supplementary Fig. 11a, b).

We calculated immune cell proportions in each sample using weighted least squares to identify key immune cells involved in the transcriptome transition from E-T1D, I-T1D to L-T1D. B cells and plasmablasts had the highest percent of total lymphocytes in E-T1D, the lowest in L-T1D, and intermediate in I-T1D (median = E-T1D-11.64%, I-T1D-8.47% and L-T1D-5.99%, *P*_*WLS*_ < 0.02). In contrast, MAIT cells showed the opposite trends in both T1D and HC groups (median = E-T1D-1.53%, I-T1D-2.53% and L-T1D-3.47%, *P*_*WLS*_ < 0.05; median = E-HC-1.79%, I-HC-3.71% and L-T1D-5.50%, *P*_*WLS*_ < 0.05) (Fig. 4d and Supplementary Fig. 11c). These trends in the proportions of B cells and MAIT cells among the three T1D subgroups were validated in an independent cohort of 15 individuals (Fig. 4e, Supplementary Fig. 12a, b). Due to the low abundance of plasmablasts in peripheral blood, we focused on B cells and MAIT cells for functional analysis. Differentially expressed genes (DEGs) were identified for each T1D subgroup in comparison with the other two subgroups, using a threshold of fold change ≥ 2 and adjusted *p-value* < 0.05. The same analysis was performed for the E-HC, I-HC, and L-HC subgroups to identify age-related transcriptional differences. DEGs in each T1D subgroup were then refined by removing those that were also upregulated in the corresponding HC subgroup to exclude age-related effects. DEG overlap analysis demonstrated that E-T1D and I-T1D shared the highest number of DEGs in both B and MAIT cells, whereas L-T1D exhibited fewer overlaps (Supplementary Fig. 11d). Functional pathway enrichment analysis revealed distinct processes across B and MAIT cells in T1D subgroups. In both cell types, E-T1D were more enriched for immune-related pathways, including NF-kappaB activation in B cells, and interferon response and interleukin-15 signaling in MAIT cells, while L-T1D were enriched for amino acid metabolism pathways, such as proteinogenic amino acid in B cells and serine family amino acid in MAIT cells. I-T1D exhibited features of both subgroups, with enrichment in pathways associated with immune and metabolism (Fig. 4f).

To analyze B cell heterogeneity in T1D groups, we re-clustered 41,675 B cells (including plasmablasts) into four subsets: naïve B cells (B_Naive), memory B cells (B_Mem), atypical B cells (B_Atypical), and plasmablasts (Plasmablast) (Supplementary Fig. 11e). B_Naive cells were most abundant in E-T1D, least in L-T1D, and intermediate in I-T1D (median = E-T1D-7.03%, I-T1D-4.91% and L-T1D-3.72%, *P*_*WLS*_ < 0.03). B_Mem and B_Atypical cells exhibited similar but not significant trends (Supplementary Fig. 11f). Due to the low abundance of B_Atypical cells and their classification as a subset of memory B cells, we combined B_Mem and B_Atypical cells into a single population (B_Mem_Atypical). DEG overlap analysis of B_Naive and B_Mem_Atypical cells showed substantial overlaps between E-T1D and I-T1D (Supplementary Fig. 11g). Pathway enrichment in B_Naive and B_Mem_Atypical cells confirmed that E-T1D was predominantly enriched for immune-related pathways, including antigen presentation, inflammatory response, and TCR signaling. L-T1D showed enrichment in metabolic pathways, particularly those involving protein and amino acid metabolism. I-T1D reflected a mix of immune and metabolic processes, aligning with its transitional phenotype (Supplementary Fig. 11h).

Collectively, these findings indicate that age-related T1D endotypes differ in both the proportion and functional state of peripheral immune cells, particularly naïve B cells, with E-T1D showing a pattern of immune activation and L-T1D showing a pattern of metabolic reprogramming.

### Multi-omics interaction network reveals DPA-mediated link between *D. invisus* and B cell activation

**Fig. 5:**
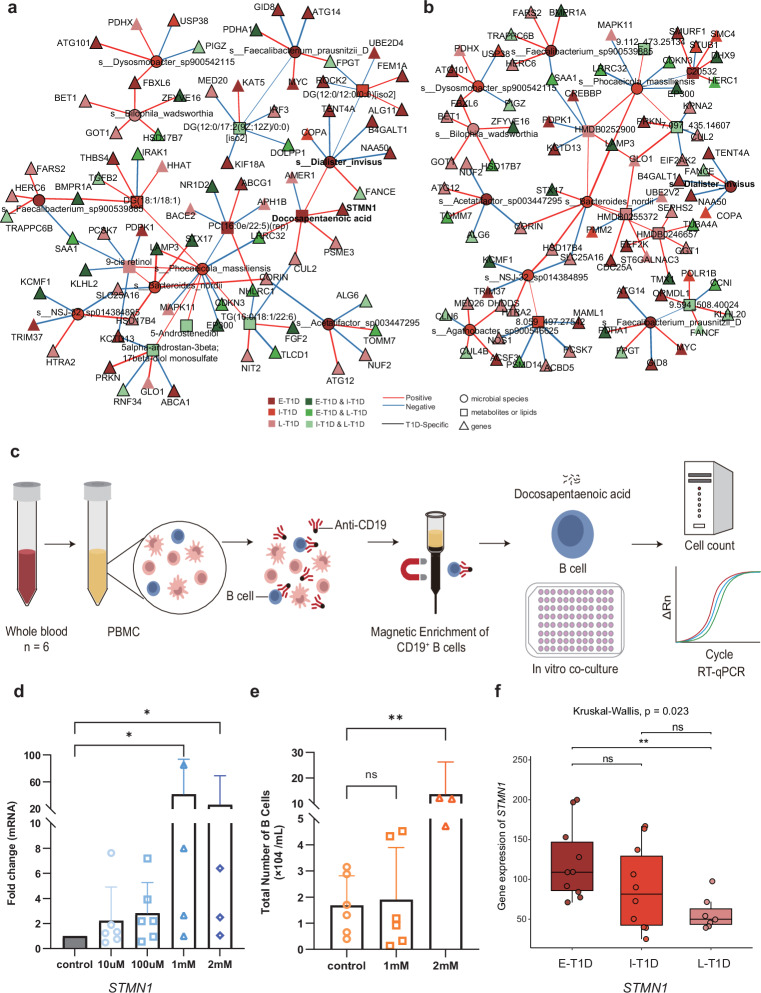
Multi-omics interaction network reveals DPA-mediated link between *D. invisus* and B cell activation. Integrated multi-omics interaction networks linking microbial species, metabolites, lipids, and genes significantly upregulated in T1D subgroups. For the core network, top 10 microbial species showing the greatest number of significant gene associations were selected as representative nodes. Endogenous (**a**) and environmental (**b**) metabolite-mediated networks were visualized separately. For each selected microbe, the top 10 correlated metabolites and, for each metabolite, the top 5 correlated genes were included. In addition, the top 5 directly correlated genes not mediated by metabolites were incorporated into the network visualization. The species *Dialister invisus*, docosapentaenoic acid (DPA), and *STMN1* are highlighted in bold. Node shape indicates data type: circles represent microbial species, squares represent metabolites or lipids, and triangles represent genes. Node color indicates the T1D subgroup in which the feature is upregulated; nodes with a black outer border represent features not upregulated in the corresponding HC subgroup. Edge color indicates correlation direction (red: positive; blue: negative), and edge width corresponds to the absolute value of the Spearman correlation coefficient. **c** Schematic illustration of the in vitro validation workflow. CD19⁺ B cells were isolated from PBMCs of 21 newly diagnosed T1D patients, enriched by magnetic sorting, and stimulated with different concentrations of DPA. Live B cell numbers and *STMN1* expression were measured in 6 patients, while culture supernatants from the other 15 patients were collected for cytokine measurement. **d** Relative expression of *STMN1* in B cells after 48-hour exposure to DPA, measured by RT-qPCR. **e** Live B cell numbers following DPA treatment at indicated concentrations. In panels d and e, data are shown as mean ± standard deviation. Statistical significance was evaluated using Kruskal-Wallis followed by Dunn’s post hoc correction. **f** Comparison of *STMN1* gene expression among the three T1D subgroups based on pseudo-bulk transcriptomic data from peripheral B cells. Expression differences were assessed using the Kruskal–Wallis test followed by pairwise Wilcoxon tests with Holm correction for multiple comparisons. Asterisks indicate *P* < 0.05 (*), *P* < 0.01 (**); “ns” indicates not significant

To identify direct interactions between the microbiota and host immune status, as well as indirect interactions mediated by metabolites or lipids, we constructed large-scale multi-omics interaction networks based on biomarkers upregulated in different T1D subgroups at various omics levels, including 65 microbiota taxa, 94 endogenous and 184 environmental metabolites, 53 lipids and 724 genes derived from the transcriptome profiles of peripheral B cells, and functionally annotated in relation to cell proliferation, inflammatory responses, and metabolic signaling (Supplementary Table 6). We constructed multi-omics networks for endogenous and environmental metabolites separately. To reduce redundancy and multicollinearity among highly correlated metabolites or lipids, we applied a conservative threshold of absolute correlation coefficient ≥ 0.9 for pairwise correlation filtering. Only the most representative metabolic features from each highly correlated cluster were retained for subsequent correlation analysis and network inference. We identified a total of 175 microbiota-metabolite/lipid interaction pairs, including 87 pairs derived from endogenous metabolites and lipids and 88 pairs from environmental metabolites. In addition, 3836 metabolite/lipid-gene interaction pairs were detected, comprising 1878 pairs from endogenous metabolites and lipids and 1958 pairs from environmental metabolites, together with 766 microbiota-gene interaction pairs. These interactions collectively formed 665 direct microbiota-gene interactions and 2608 significant microbiota-metabolite/lipid-gene triadic interactions, consisting of 1293 pairs from endogenous metabolites and lipids, and 1315 pairs from environmental metabolites. (Fig. 5a, b, Supplementary Table 7).

In the multi-omics network mediated by endogenous metabolites or lipids, 64.62% (*n* = 42) of connected microbial species were upregulated in I-T1D and were significantly associated with triacylglycerol (20.00%, *n* = 13), urine albumin creatinine ratio (21.54%, *n* = 14), age at diagnosis (21.54%, *n* = 14), and fasting C-peptide (10.77%, *n* = 7). Among the connected metabolites or lipids, 48.00% (*n* = 60) were upregulated in E-T1D, primarily significantly associated with high-density lipoprotein cholesterol (48.00%, *n* = 60), triacylglycerol (52.80%, *n* = 66), and age at diagnosis (97.60%, *n* = 122). Notably, 58.40% (*n* = 73) of them showed significant correlation with fasting C-peptide levels, while 13.60% (*n* = 17) also correlated with thyroid peroxidase antibody levels. Among the connected genes, most were upregulated in E-T1D (53.82%, *n* = 345) and L-T1D (58.35%, *n* = 374), primarily associated with urine albumin-creatinine ratio (16.07%, *n* = 103), age at diagnosis (13.42%, *n* = 86), HbA1c (11.70%, *n* = 75), thyroid peroxidase antibodies (7.33%, *n* = 47), high-density lipoprotein cholesterol (8.89%, *n* = 57), and total cholesterol (4.99%, *n* = 32). In the multi-omics network mediated by environmental metabolites, the distribution of connected microbiota within T1D subgroups and their correlation with clinical variables aligned with those observed in the endogenous network. Among the connected metabolites, 57.46% (*n* = 77) were upregulated in L-T1D, primarily significantly associated with high-density lipoprotein cholesterol (27.61%, *n* = 37), triacylglycerol (29.1%, *n* = 39), lymphocyte count (20.90% *n* = 28), and age at diagnosis (96.27%, *n* = 129). And 57.46% (*n* = 77) of them showed significant correlation with fasting C-peptide levels, while 7.46% (*n* = 10) demonstrated significant correlation with thyroid peroxidase antibodies. Among the connected genes, most were upregulated in E-T1D (53.75%, *n* = 351) and L-T1D (59.11%, *n* = 386), primarily associated with urine albumin-creatinine ratio (15.77%, *n* = 103), age at diagnosis (13.32%, *n* = 87), HbA1c (10.87%, *n* = 71), thyroid peroxidase antibodies (7.50%, *n* = 49), high-density lipoprotein cholesterol (8.27%, *n* = 54), and total cholesterol (5.05%, *n* = 33) (Supplementary Table 8).

Among all microbiota taxa identified at the species level, the species *Dialister invisus* was well connected and showed significant correlations with the largest number of genes (*n* = 26), where correlations with *ROCK2*, *STMN1*, and *ALG11* could be mediated by metabolites, with the strongest correlation observed with *STMN1*. *D. invisus* is specifically upregulated in the E-T1D subgroup and negatively correlated with age at diagnosis, showing significant correlations only with the endogenous metabolites DG(12:0/12:0/0:0) [iso2] and Docosapentaenoic acid (DPA). It participated in 18 triadic interactions involving these two metabolites, including the *D. invisus*-DPA-*STMN1* triad. DPA was also upregulated in the E-T1D subgroup and negatively correlated with diagnostic age. Although DPA was not a T1D-specific biomarker, which was also upregulated in the E-HC subgroup compared with the other two HC subgroups, its abundance in T1D was significantly higher than in the HC groups (FC = 1.33, *P* < 1e-4). To validate the effect of DPA on the peripheral immune environment, we stimulated B cells isolated from PBMCs with different concentrations of DPA in vitro (Fig. 5c). We found that under high concentrations of DPA stimulation (1 mM, 2 mM), B cells proliferated and upregulated the expression of the *STMN1* gene, which was a regulator of cell proliferation and differentiation^23,24^ (Fig. 5d, e). Accordant with these findings, transcriptomic profiling also revealed that *STMN1* expression was highest in the E-T1D subgroup (Fig. 5f). The results of the in vitro experiments were consistent with those from the multi-omics interaction network and peripheral transcriptomes, which showed a significant positive correlation between DPA abundance and *STMN1* gene expression in B cells, as well as the highest proportion of peripheral B cells in the E-T1D group. Moreover, the concentrations of interleukin-6 (IL-6) and IgM in the culture supernatant were markedly increased following DPA stimulation (Supplementary Fig. 13a, b), indicating enhanced activation and antibody secretion of B cells.

These findings identify a potential regulatory relationship among *D. invisus*, DPA, and *STMN1*, providing integrative evidence for a link between microbial, metabolic, and immune alterations in age-related T1D endotypes.

## Discussion

Type 1 diabetes (T1D) is a heterogeneous autoimmune disease, with age at onset influencing disease presentation, progression, and immune regulation.^6^ Previous studies have highlighted the critical role of the gut microbiome and its metabolites in regulating host metabolic and immune responses, with potential implications for various diseases.^25–29^ In this study, we profiled age-related endotypes of T1D by integrating microbiome, metabolome, lipidome, and transcriptome data. Our aim was to identify subgroup-specific biomarkers and clarify the molecular mechanisms underlying early-onset (E-T1D), intermediate-onset (I-T1D), and late-onset (L-T1D) T1D. Building on this framework, we identified, for the first time in the context of T1D, direct relationships between the microbiota and the peripheral immune environment, as well as indirect relationships mediated by metabolites and lipids. By constructing multi-omics interaction networks, we sought to uncover regulatory relationships between microbiota, metabolites, lipids, and host genes that could contribute to the onset and progression of T1D across different age groups.

Consistent with Finnish Pediatric Diabetes Register study,^11^ we observed that HLA high-risk genotypes were most frequent in E-T1D and lowest in L-T1D. We also found that fasting C-peptide levels increased with age at diagnosis, accordant with previous findings.^10,11^ We identified dyslipidemia in the L-T1D subgroup, including elevated total cholesterol and reduced high-density lipoprotein cholesterol, which are commonly associated with poor glycemic control^30^ and suggest more metabolic disturbances in this group. Previous studies have shown that thyroid peroxidase antibodies, a characteristic marker of autoimmune thyroid disease, commonly coexist with T1D.^31^ Similarly, we observed higher levels in T1D patients than in healthy controls, with a non-significant upward trend from E-T1D to L-T1D.

Across the microbiome, *Acetatifactor*, *Firmicutes A*, and *Bacteroidaceae* were enriched in E-T1D, I-T1D, and L-T1D, respectively, highlighting age-specific microbial signatures. The genus *Acetatifactor*, belonging to the *Firmicutes*, is associated with lipopolysaccharide production and inflammation.^32^ Studies have shown that a reduction in the relative abundance of *Acetatifactor* in type 2 diabetes mouse models is linked to a decrease in blood glucose levels.^33^ In the E-T1D subgroup, the most significantly enriched pathway was pentose and glucuronate interconversions, which has been implicated in the development of diabetic nephropathy,^34^ a common renal complication of T1D. And phenylalanine metabolism was the most enriched pathway in the L-T1D subgroup. Elevated phenylalanine levels have been previously reported in both children with T1D^35^ and in T1D patients with metabolic dysfunction-associated steatotic liver disease,^36^ aligning with the dyslipidemia profile observed in this subgroup.

Distinct metabolites further characterized each subgroup. Elevated pipecolic acid and phthalic acid in E-T1D may indicate immune and environmental triggers of early disease onset.^37,38^ N-acetylcitrulline, an N-acetyl-amino acid derivative of citrulline enriched in L-T1D, is involved in arginine-citrulline metabolism and reflects alterations in amino acid acetylation.^39^ These results suggest an association between environmental factors and the autoimmunity of E-T1D, while L-T1D appears to be more closely related to metabolic disturbances. Lipidomic analysis identified LPA (16:1), TG (16:0/18:2/18:3), and TG (18:0/18:1/18:1) as the most perturbed lipids in E-, I-, and L-T1D, respectively. Elevated in LPA levels have been observed in STZ-induced diabetes in rats, where Oxi-LDL/LPA/LPAR1/BACE1 signaling cascade promotes β-cell injury and oxidative stress, implicating LPA as a potential mediator of early-onset T1D pathogenesis.^40^ In contrast, triglyceride alterations in I- and L-T1D resemble metabolic profiles observed in newly diagnosed type 2 diabetes,^41,42^ underscoring shared metabolic mechanisms in late-onset T1D. These findings underscore distinct metabolic trajectories across age-related T1D endotypes.

At the transcriptomic level, the proportion of B cells, particularly naïve B cells, declined progressively across E-T1D, I-T1D, and L-T1D. This aligns with the immune cell infiltration patterns observed in islets, where younger T1D patients (<7 years old) exhibit higher levels of CD20^+^ B cell infiltration in inflamed islets.^8,9^ This suggests that B cells, primarily naive B cells, may have the potential to distinguish whether T1D occurs early or late. A recent study by Luo et al. similarly reported an increased proportion of peripheral naïve B cells in newly diagnosed T1D patients within 250 days of diagnosis, supporting that our participants were also in the early stage of disease.^43^ In addition to differences in cell proportions, B cells exhibit distinct functional changes across age-related endotypes of T1D, with immune responses dominant in E-T1D, metabolism dominant in L-T1D, and I-T1D exhibiting mixed characteristics. Both immune and metabolism are key mechanisms in T1D pathogenesis,^20,21,44,45^ suggesting that age-related T1D endotypes may involve distinct pathogenic processes. These differences in the proportion and function of immune cells may explain the different disease phenotypes associated with age-related T1D endotypes, with E-T1D typically being more aggressive and L-T1D tending to be milder.

Multi-omics correlation networks further revealed that most endogenous and environmental metabolites correlated with age at diagnosis and fasting C-peptide, suggesting their close involvement in T1D autoimmune processes. Notably, *Dialister invisus*, previously linked to prediabetic risk,^46^ and we found it was enriched in the E-T1D and strongly connected to cell proliferation, including *STMN1*, *ROCK2*, and *TENT4A*. It was also associated with genes linked to interferon signaling, such as *NUP58* and *PSMD14*, and with genes involved in antigen presentation, including *ACTR10* and *ATG10*. The involvement of these genes in immune regulation and β-cell autoimmunity^44,45^ underscores the potential pathogenic relevance of *D. invisus* in T1D. Among the characteristic metabolites in T1D subgroups, *D.invisus* showed the strongest correlation with DPA and formed a triadic interaction with *STMN1*. DPA was upregulated in E-T1D and has been reported to be upregulated in overweight women with early-onset gestational diabetes mellitus.^47^ We validated through in vitro experiments that higher concentrations of docosapentaenoic acid can stimulate the proliferation of peripheral blood-derived B cells and upregulate the expression of *STMN1*. Moreover, we observed increased secretion of IL-6 and IgM in the culture supernatant. These cytokines are closely associated with inflammatory responses and autoantibody production in T1D,^48,49^ which indicates that DPA stimulation promotes immune activation of B cells. *STMN1* is a key gene involved in mitosis^23^ and has been reported to exhibit elevated expression in lymphoblastoid cell lines and B cells infected with Epstein-Barr virus compared to resting B cells,^50^ which is hypothesized to potentially induce T1D through molecular mimicry.^51^ We found that *STMN1* expression is specifically upregulated in the E-T1D subgroup. Combined with the highest peripheral B cell proportion observed in E-T1D, this reveals a potential pathogenic mechanism, that the upregulation of *D. invisus* in E-T1D may upregulate *STMN1* expression via docosapentaenoic acid, thereby promoting B cell proliferation and activating autoimmune responses. This strategy, combining multi-omics analysis and in vitro experiments, can help us identify key pathogenic molecules associated with T1D.

This study has several limitations. First, the cross-sectional design of this study restricts causal interpretation. While most T1D individuals included had a disease duration of less than 3 years, longitudinal studies are needed to evaluate whether these findings indeed contribute to the early onset of T1D. Second, the relatively small sample size of scRNA-seq data limits the statistical power of the corresponding results. Finally, in the multi-omics interaction network, we only partially validated the regulatory relationships between the metabolite and the gene, and correlations between the microbiota and metabolites require further investigation.

In conclusion, this study reveals distinct microbial, metabolic, lipid, and immune signatures among age-related T1D endotypes. The E-T1D subgroup exhibited pronounced immune activation, the L-T1D subgroup showed metabolic predominance, and the I-T1D subgroup displayed intermediate characteristics. Functional validation supports inter-omics regulatory links from multi-omics correlation networks, highlighting the *D. invisus*-DPA-*STMN1* triad as a potential immune-metabolic target in T1D.

## Materials and methods

### Subject recruitment and sample collection

The T1D China Registry Study (ChiCTR2000034642) recruited T1D individuals at multiple centers in China from 2019 to 2024. This study included 108 newly diagnosed T1D individuals (diabetes duration ≤3 years) and categorized them into three subgroups based on age at diagnosis: early-onset T1D (E-T1D, <7 years), intermediate-onset T1D (I-T1D, 7–12 years), and late-onset T1D (L-T1D, ≥13 years). Next, individuals with T1D were matched by age and sex to healthy control subjects in a 2:1 ratio (2 individuals with T1D to 1 healthy control subject), resulting in 56 healthy individuals who were included as control subjects. Healthy controls were recruited and divided into three subgroups based on age: healthy controls under 7 years (E-HC), healthy controls aged 7–12 years (I-HC), and healthy controls aged ≥13 years (L-HC). A total of 69 fecal samples from T1D patients and 56 from healthy controls were collected in sterile containers and stored at −80 °C after transport to the laboratory. Blood samples from 108 T1D patients and 56 healthy controls were collected for metabolic examination and preserved at −80 °C. From donors with matched microbiome, metabolome, and lipidome data, we selected a total of 54 participants, including 27 T1D patients and 27 age- and sex-matched healthy controls. Whole blood was collected to isolate PBMCs, followed by single-cell RNA transcriptome sequencing. DPA stimulation assays were performed using PBMCs from an independent cohort of 21 individuals with T1D, and quantitative real-time PCR, cell counting, and cytokine measurements were conducted after 48 h of incubation. Among these, PBMCs from 15 individuals were additionally analyzed by flow cytometry.

### Ethics statement

The Ethics Committee of The First Affiliated Hospital of University of Science and Technology of China permitted this study (No. 2019KY027). Our study was conducted based on the Declaration of Helsinki. Every participant provided written informed consent.

### Fecal metagenomic analysis

In this study, microbiome metagenomic sequencing of fecal samples was conducted on a desktop genetic sequencer (DNBSEQ platform) employing Rolling Circle Amplification sequencing. Each amplification round in Rolling Circle Amplification sequencing utilized the template from the initial round to linearly amplify, thereby mitigating cumulative sequencing errors. Subsequently, sequencing data underwent quality control, sequence assembly, and gene prediction to obtain genetic information. Reads containing 10% uncertain bases, sequencing adapter sequences, and low-quality bases were filtered out for data quality control. Assembly of samples was performed de novo using MEGAHIT software, with assembled sequences shorter than 200 bp discarded. MetaGeneMark was employed for de novo prediction of metagenomic genes, followed by redundancy removal using CD-HIT software across predicted genes from each sample. Functional annotation of non-redundant genes was typically achieved using Diamond’s BLASTP against databases including BacMet, CARD, KEGG, eggNOG, COG, Swiss-Prot, and CAZy. Genes were annotated to species and functional databases to understand microbial community composition, community structure and functional pathways. Finally, differences in species composition and community functions between different samples were compared using the Omiscribe website.

Beta diversity of the gut microbiome was calculated using the Euclidean distance based on genus-level relative abundance data. Group differences were assessed with the Kruskal–Wallis test followed by pairwise Wilcoxon rank-sum tests. All *p*-values were adjusted for multiple comparisons using the Holm correction method.

### Serum metabolomics and serum lipidomics analysis

The serum metabolites and serum lipids were detected by untargeted liquid chromatography-tandem mass spectrometry. For enhancing the scope of lipid detection, the high-resolution mass spectrometer Q Exactive (Thermo Fisher Scientific, USA) was run not only in negative but also in positive ion modes. Subsequently, we utilized the Omiscribe platform for data analysis. We conducted OPLS-DA to examine the distribution of plasma metabolites and lipids. Differential metabolites and lipids were screened in pairwise group comparisons using Student's t-test, followed by Benjamini-Hochberg correction. Metabolites and lipids meeting the criteria of VIP ≥ 1, fold change ≥ 1.2 or ≤ 0.83, and q-value < 0.05 were considered differential. Group-wise comparisons of specific metabolites were performed using the Kruskal–Wallis test followed by pairwise Wilcoxon rank-sum tests with Holm correction for multiple comparisons.

### Single-cell RNA analysis of peripheral blood mononuclear cells

PBMCs were processed using the 10x Genomics Chromium Single Cell 3’ Reagent Kits v3.1, and libraries were sequenced on the Illumina NovaSeq 6000 platform. Reads were aligned to the GRCh38 (hg38) reference genome and transcriptome using Cell Ranger (v7.1.0), and count matrices were merged across samples. Data preprocessing and downstream analysis were performed using Scanpy (v1.10.3). Genes expressed in fewer than 3 cells and cells with > 6% mitochondrial gene content were excluded. DoubletDetection (v4.2) was applied with default settings to remove doublets, and cells expressing 200-6000 genes were retained for normalization and logarithmic transformation.

Dimensionality reduction and clustering were performed by constructing a neighborhood graph (n_neighbors = 15, n_pcs = 40), followed by Leiden clustering (resolution = 1.3). Cell types were annotated manually using reported marker genes.^52,53^ The proportion of each immune cell type was calculated per sample. A weighted least squares model was then used to assess differences between groups, with the total number of cells per sample used as weights, and p-values adjusted using the Bonferroni method. Model assumptions of homoscedasticity and normality of residuals were tested using the Breusch-Pagan and Shapiro-Wilk tests, respectively. DEGs were determined in Scanpy using the Wilcoxon rank-sum test, and p-values were adjusted for multiple testing using the Benjamini-Hochberg false discovery rate method. Genes with adjusted *P* < 0.05 and log₂ fold change ≥ 1 were considered significant. DEG sets were subjected to pathway enrichment analysis using Metascape.^54^

### Partial least squares discriminant analysis

Partial least squares discriminant analysis was performed separately for metagenomic, metabolomic, lipidomic, and transcriptomic datasets using the *ropls* package in R. All variables were log transformed to stabilize variance and subsequently Pareto scaled to reduce the impact of dominant features while maintaining data structure. For the transcriptomic data, gene expression counts were aggregated into sample-level pseudo-bulk profiles and normalized to log-transformed counts per million to account for sequencing depth differences.

Each model was built with two predictive components, allowing visualization in a two-dimensional latent variable space. Model robustness was assessed through 1000 permutation tests. The number of cross-validation folds was determined according to sample size so that every group contained at least two samples per fold. Five-fold cross-validation was used for the metagenomic and transcriptomic datasets, whereas seven-fold cross-validation was applied to the lipidomic and metabolomic datasets.

Within each omics layer, three separate analyses were conducted, including comparisons among the three subgroups of T1D, among the three subgroups of HC, and across all six subgroups combined. In the metabolomic layer, endogenous and environmental metabolites were analyzed separately.

The resulting score plots displayed the distribution of samples along the first two latent variables, with each sample colored according to group. Ellipses corresponding to a 95% confidence region were drawn based on multivariate normal estimation to illustrate within-group variability and inter-group separation. The percentages shown on the x- and y-axes represent the proportion of total variance explained by the first and second latent variables, respectively.

### Flow cytometry assay

Cryopreserved PBMCs were thawed, washed, and resuspended in staining buffer. Cells were first incubated with Fixable Viability Dye 777 (APC-Cy7/Zombie NIR, 637–780 nm; STARTER, cat. no. S0D0026) for live/dead discrimination, followed by surface staining for 20 min at 4°C in the dark with the following monoclonal antibodies: FITC-anti-CD3 (BioLegend, cat. no. 300406), BV605-anti-CD19 (BD Pharmingen, cat. no. 562654), BV421-anti-CD20 (BD Pharmingen, cat. no. 740002), PE-anti-CD8α (BioLegend, cat. no. 301008), PE-Cy7-anti-TCR Vα7.2 (BioLegend, cat. no. 351711), and AF700-anti-CD161 (BioLegend, cat. no. 339941). Antibodies were titrated to optimal working concentrations prior to staining. MAIT cells were identified as CD3⁺Vα7.2⁺CD161⁺ populations, and B cells were gated as CD3^-^CD19⁺CD20⁺ cells. Differences in cell population proportions between groups were assessed using the Kruskal-Wallis test followed by pairwise Wilcoxon tests with Holm correction for multiple comparisons.

### Integrated multi-omics network analysis across age-related subgroups

We constructed multi-omics interaction networks using gut microbial species, endogenous and environmental serum metabolites, serum lipids, and gene expression profiles. For each T1D subgroup, features significantly upregulated relative to the remaining groups were included. Each omics layer underwent tailored preprocessing prior to integration. Gut microbial abundance data were normalized using the centered log-ratio transformation after total-sum scaling. Serum metabolite and lipid intensity matrices were log-transformed, standardized by z-score scaling, and filtered to remove highly collinear features with pairwise correlation coefficients ≥ 0.9, thereby reducing redundancy among correlated variables. Gene expression profiles from peripheral B cells were normalized by library size and transformed into log scale after scaling to one million total counts per sample.

Samples from healthy controls were excluded to focus on T1D-specific associations. For each omics pair (microbiome-metabolome/lipidome, metabolome/lipidome-transcriptome, and microbiome-transcriptome), matrices were matched based on sample identifiers. Pairwise dependencies between omics layers were estimated using Gaussian copula graphical modeling with graphical lasso regularization and the Stability Approach to Regularization Selection to infer sparse conditional dependencies. To further ensure robustness, Spearman’s rank correlations were calculated for all paired features, and p-values were adjusted for multiple testing using the Benjamini-Hochberg procedure. Only associations that remained significant under both methods ( q-value < 0.05) were retained as high-confidence edges.

Networks were visualized with Cytoscape (v3.10.3). For the core network, top 10 microbial species with the greatest number of significant gene associations were selected as representative nodes. Endogenous and environmental metabolite-mediated networks were constructed separately. For each microbe, the top 10 correlated metabolites and, for each metabolite, the top 5 correlated genes were included. Additionally, the top 5 directly correlated genes not mediated by metabolites were incorporated into the visualization.

### In vitro stimulation of CD19⁺ B cells with docosapentaenoic acid

PBMCs were isolated from whole blood of 6 newly diagnosed T1D patients (age ≤18 years, disease duration < 1 year). CD19⁺ B cells were purified via positive magnetic selection using the MojoSort™ Human CD19 Selection Kit (BioLegend) following the manufacturer’s instructions. Purified B cells were cultured in X-VIVO™ 15 medium (Lonza) containing docosapentaenoic acid (APE×BIO) at gradient concentrations. After 48 h of incubation, live cell counts were assessed using a Countstar Automated Cell Counter (ALIT Life Science). Total RNA was extracted using the RNA-Quick Purification Kit (YiShan Biotech, Shanghai, China), and reverse transcription was conducted with 5× PrimeScript™ RT Master Mix (Takara). Quantitative real-time PCR was performed on a LightCycler® 96 System (Roche) using SYBR Green qPCR Mix (TOLOBIO, China), with GAPDH as the reference gene for normalization. Experimental results were visualized using GraphPad Prism software (v10). Comparisons of *STMN1* gene expression and total B-cell counts among different concentrations of docosapentaenoic acid were performed using the Kruskal-Wallis test followed by Dunn’s post hoc correction for multiple comparisons. The concentrations of cytokines in the supernatants were quantified using enzyme-linked immunosorbent assay kits according to the manufacturers’ instructions. The following kits were used: BAFF (Absin, cat. no. ABS5510663; lot JA0CKAC8EL), IL-6 (Absin, cat. no. ABS510003; lot J0724R01), and IgM (Absin, cat. no. ABS551024; lot JA0CKAC1EL). Absorbance was measured at 450 nm using a microplate reader, and cytokine concentrations were calculated based on standard curves generated for each analyte. Group differences in cytokine concentrations were evaluated using the Kruskal-Wallis test followed by pairwise Wilcoxon tests with Holm correction for multiple comparisons.

## Supplementary information

Supplementary materials for Multi-omics reveals microbiota, metabolite, and immunological heterogeneity of age-related endotypes in type 1 diabetes

Supplementary Table 6

Supplementary Table 7

Supplementary Table 8

Supplementary Table 9

Supplementary Table 10

## Data Availability

The metagenomic data have been deposited in the SRA database under accession number PRJNA1285275, and the metabolomic and lipidomic data have been deposited in the National Genomics Data Center under accession number PRJCA049399. The single-cell RNA-seq data have been deposited in NCBI’s Gene Expression Omnibus^55^ and are accessible through GEO Series accession number GSE316337 (https://www.ncbi.nlm.nih.gov/geo/query/acc.cgi?acc=GSE316337). All datasets have been released.

## References

[1] Ilonen, J., Lempainen, J. & Veijola, R. The heterogeneous pathogenesis of type 1 diabetes mellitus. *Nat. Rev. Endocrinol.* **15**, 635–650 (2019). 31534209 10.1038/s41574-019-0254-y

[2] Song, Y., Li, J. & Wu, Y. Evolving understanding of autoimmune mechanisms and new therapeutic strategies of autoimmune disorders. *Signal Transduct. Target Ther.* **9**, 263 (2024). 39362875 10.1038/s41392-024-01952-8 PMC11452214

[3] Katsarou, A. et al. Type 1 diabetes mellitus. *Nat. Rev. Dis. Prim.* **3**, 17016 (2017). 28358037 10.1038/nrdp.2017.16

[4] Weng, J. et al. Incidence of type 1 diabetes in China, 2010-13: population based study. *BMJ* **360**, j5295 (2018). 29298776 10.1136/bmj.j5295 PMC5750780

[5] Gregory, G. A. et al. Global incidence, prevalence, and mortality of type 1 diabetes in 2021 with projection to 2040: a modelling study. *Lancet Diab. Endocrinol.* **10**, 741–760 (2022). 10.1016/S2213-8587(22)00218-2 36113507

[6] Redondo, M. J. & Morgan, N. G. Heterogeneity and endotypes in type 1 diabetes mellitus. *Nat. Rev. Endocrinol.* **19**, 542–554 (2023). 37337007 10.1038/s41574-023-00853-0

[7] Battaglia, M. et al. Introducing the endotype concept to address the challenge of disease heterogeneity in type 1 diabetes. *Diab. Care* **43**, 5–12 (2020). 10.2337/dc19-0880 PMC6925574 31753960

[8] Arif, S. et al. Blood and islet phenotypes indicate immunological heterogeneity in type 1 diabetes. *Diabetes* **63**, 3835–3845 (2014). 24939426 10.2337/db14-0365 PMC4207393

[9] Leete, P. et al. Differential insulitic profiles determine the extent of β-cell destruction and the age at onset of type 1 diabetes. *Diabetes* **65**, 1362–1369 (2016). 26858360 10.2337/db15-1615

[10] Leete, P. et al. Studies of insulin and proinsulin in pancreas and serum support the existence of aetiopathological endotypes of type 1 diabetes associated with age at diagnosis. *Diabetologia* **63**, 1258–1267 (2020). 32172310 10.1007/s00125-020-05115-6 PMC7228905

[11] Parviainen, A. et al. Heterogeneity of type 1 diabetes at diagnosis supports existence of age-related endotypes. *Diab. Care* **45**, 871–879 (2022). 10.2337/dc21-1251 35147706

[12] Inshaw, J. R. J., Cutler, A. J., Crouch, D. J. M., Wicker, L. S. & Todd, J. A. Genetic variants predisposing most strongly to type 1 diabetes diagnosed under age 7 years lie near candidate genes that function in the immune system and in pancreatic β-cells. *Diab. Care* **43**, 169–177 (2020). 10.2337/dc19-0803 PMC6925581 31558544

[13] Rawshani, A. et al. Excess mortality and cardiovascular disease in young adults with type 1 diabetes in relation to age at onset: a nationwide, register-based cohort study. *Lancet* **392**, 477–486 (2018). 30129464 10.1016/S0140-6736(18)31506-X PMC6828554

[14] Tobias, D. K. et al. Second international consensus report on gaps and opportunities for the clinical translation of precision diabetes medicine. *Nat. Med.* **29**, 2438–2457 (2023). 37794253 10.1038/s41591-023-02502-5 PMC10735053

[15] Powers, A. C. Type 1 diabetes mellitus: much progress, many opportunities. *J. Clin. Invest.* **131**, e142242 (2021). 10.1172/JCI142242 PMC8262558 33759815

[16] Balasubramanyam, A. Defining and classifying new subgroups of diabetes. *Annu. Rev. Med.* **72**, 63–74 (2021). 33064971 10.1146/annurev-med-050219-034524

[17] van Belle, T. L., Coppieters, K. T. & von Herrath, M. G. Type 1 diabetes: etiology, immunology, and therapeutic strategies. *Physiol. Rev.* **91**, 79–118 (2011). 21248163 10.1152/physrev.00003.2010

[18] Vatanen, T. et al. The human gut microbiome in early-onset type 1 diabetes from the TEDDY study. *Nature* **562**, 589 (2018). 30356183 10.1038/s41586-018-0620-2 PMC6296767

[19] Mokhtari, P., Jambal, P., Metos, J. M., Shankar, K. & Babu, P. V. A. Microbial taxonomic and functional shifts in adolescents with type 1 diabetes are associated with clinical and dietary factors. *Ebiomedicine* **93**, 104641 (2023). 10.1016/j.ebiom.2023.104641 PMC10272319 37290262

[20] Oresic, M. et al. Dysregulation of lipid and amino acid metabolism precedes islet autoimmunity in children who later progress to type 1 diabetes. *J. Exp. Med.* **205**, 2975–2984 (2008). 19075291 10.1084/jem.20081800 PMC2605239

[21] Yuan, X. X. et al. Functional and metabolic alterations of gut microbiota in children with new-onset type 1 diabetes. *Nat. Commun.* **13**, 6356 (2022). 10.1038/s41467-022-33656-4 PMC9606127 36289225

[22] Honardoost, M. A. et al. Systematic immune cell dysregulation and molecular subtypes revealed by single-cell RNA-seq of subjects with type 1 diabetes. *Genome Med.* **16**, 45 (2024). 10.1186/s13073-024-01300-z PMC10976681 38539228

[23] Cuijpers, S. A. G. et al. Chromokinesin KIF4A teams up with stathmin 1 to regulate abscission in a SUMO-dependent manner. *J Cell Sci.* **133**, jcs248591 (2020). 10.1242/jcs.248591 PMC7390632 32591481

[24] Liu, R. et al. STNM1 in human cancers: role, function and potential therapy sensitizer. *Cell Signal* **109**, 110775 (2023). 37331415 10.1016/j.cellsig.2023.110775

[25] Wu, J., Wang, K., Wang, X., Pang, Y. & Jiang, C. The role of the gut microbiome and its metabolites in metabolic diseases. *Protein Cell* **12**, 360–373 (2021). 33346905 10.1007/s13238-020-00814-7 PMC8106557

[26] Kolli, U. et al. Multi-omics analysis revealing the interplay between gut microbiome and the host following opioid use. *Gut Microbes* **15**, 2246184 (2023). 10.1080/19490976.2023.2246184 PMC10448978 37610102

[27] Qian, X. H. et al. Multi-omics data reveals aberrant gut microbiota-host glycerophospholipid metabolism in association with neuroinflammation in APP/PS1 mice. *Gut Microbes* **15**, 2282790 (2023). 10.1080/19490976.2023.2282790 PMC10730179 37992400

[28] Shen, Y. et al. Alterations in gut microbiome and metabolomics in chronic hepatitis B infection-associated liver disease and their impact on peripheral immune response. *Gut Microbes* **15**, 2155018 (2023). 10.1080/19490976.2022.2155018 PMC9757487 36519342

[29] Zhao, Q. et al. Intestinal dysbiosis exacerbates the pathogenesis of psoriasis-like phenotype through changes in fatty acid metabolism. *Signal Transduct Target Ther.* **8**, 40 (2023). 10.1038/s41392-022-01219-0 PMC9884668 36710269

[30] Guy, J. et al. Lipid and lipoprotein profiles in youth with and without type 1 diabetes The SEARCH for diabetes in youth case-control study. *Diab. Care* **32**, 416–420 (2009). 10.2337/dc08-1775 PMC2646019 19092167

[31] Jonsdottir, B. et al. Thyroid and islet autoantibodies predict autoimmune thyroid disease at type 1 diabetes diagnosis. *J. Clin. Endocr. Metab.* **102**, 1277–1285 (2017). 28388722 10.1210/jc.2016-2335 PMC5460724

[32] Wu, Y. et al. Preventive effects of polysaccharides from Physalis alkekengi L. on dietary advanced glycation end product-induced insulin resistance in mice associated with the modulation of gut microbiota. *Int J. Biol. Macromol.* **204**, 204–214 (2022). 35108598 10.1016/j.ijbiomac.2022.01.152

[33] Ge, X. D. et al. Porous starch microspheres loaded with luteolin exhibit hypoglycemic activities and alter gut microbial communities in type 2 diabetes mellitus mice. *Food Funct.* **16**, 54-70 (2025). 10.1039/d4fo02907k 39377562

[34] Sandholm, N. et al. The genetic landscape of renal complications in type 1 diabetes. *J. Am. Soc. Nephrol.* **28**, 557–574 (2017). 27647854 10.1681/ASN.2016020231 PMC5280020

[35] Galderisi, A. et al. Metabolomics reveals new metabolic perturbations in children with type 1 diabetes. *Pediatr. Diab.* **19**, 59–67 (2018). 10.1111/pedi.12524 28401628

[36] Taiwo, A. et al. Metabolite perturbations in type 1 diabetes associated with metabolic dysfunction-associated steatotic liver disease. *Front Endocrinol.* **16**, 1500242 (2025). 10.3389/fendo.2025.1500242 PMC12188458 40568565

[37] Ye, L. et al. Aflatoxin B1-induced liver pyroptosis is mediated by disturbing the gut microbial metabolites: the roles of pipecolic acid and norepinephrine. *J. Hazard Mater.* **474**, 134822 (2024). 10.1016/j.jhazmat.2024.134822 38850943

[38] Castro-Correia, C. et al. Phthalates and type 1 diabetes: is there any link? *Environ. Sci. Pollut. R.* **25**, 17915–17919 (2018). 10.1007/s11356-018-1997-z PMC6028856 29680886

[39] Xu, Y., Labedan, B. & Glansdorff, N. Surprising arginine biosynthesis: a reappraisal of the enzymology and evolution of the pathway in microorganisms. *Microbiol. Mol. Biol. Rev.* **71**, 36–47 (2007). 17347518 10.1128/MMBR.00032-06 PMC1847373

[40] Abo El-Magd, N. F., Ramadan, N. M. & Eraky, S. M. The ameliorative effect of bromelain on STZ-induced type 1 diabetes in rats through Oxi-LDL/LPA/LPAR1 pathway. *Life Sci.* **285**, 119982 (2021). 10.1016/j.lfs.2021.119982 34592232

[41] Barranco-Altirriba, M. et al. Lipidome characterisation and sex-specific differences in type 1 and type 2 diabetes mellitus. *Cardiovasc. Diabetol.* **23**, 109 (2024). 10.1186/s12933-024-02202-5 PMC10981308 38553758

[42] Schön, M. et al. Intramyocellular triglyceride content during the early course of type 1 and type 2 diabetes. *Diabetes* **72**, 1483–1492 (2023). 37478166 10.2337/db23-0353 PMC10545555

[43] Luo, S. W. et al. TCL1A in naïve B cells as a therapeutic target for type 1 diabetes. *Ebiomedicine* **113**, 105593 (2025). 10.1016/j.ebiom.2025.105593 PMC11872515 39946833

[44] Herold, K. C. et al. The immunology of type 1 diabetes. *Nat. Rev. Immunol.* **24**, 435–451 (2024). 38308004 10.1038/s41577-023-00985-4 PMC7616056

[45] Bluestone, J. A., Buckner, J. H. & Herold, K. C. Immunotherapy: building a bridge to a cure for type 1 diabetes. *Science* **373**, 510–515 (2021). 34326232 10.1126/science.abh1654

[46] Maffeis, C. et al. Association between intestinal permeability and faecal microbiota composition in Italian children with beta cell autoimmunity at risk for type 1 diabetes. *Diab. Metab. Res. Rev.* **32**, 700–709 (2016). 10.1002/dmrr.2790 26891226

[47] Masalin, S. et al. Analysis of early-pregnancy metabolome in early- and late-onset gestational diabetes reveals distinct associations with maternal overweight. *Diabetologia* **67**, 2539–2554 (2024). 39083240 10.1007/s00125-024-06237-x PMC11519293

[48] Li, J. et al. Serum IL-17A concentration and a IL17RA single nucleotide polymorphism contribute to the risk of autoimmune type 1 diabetes. *Diabetes Metab. Res. Rev.* **38**, e3547 (2022). 10.1002/dmrr.3547 35583128

[49] Hundhausen, C. et al. Enhanced T cell responses to IL-6 in type 1 diabetes are associated with early clinical disease and increased IL-6 receptor expression. *Sci. Transl. Med.* **8**, 356ra119 (2016). 27629486 10.1126/scitranslmed.aad9943 PMC5125295

[50] Baik, S. Y. et al. Identification of stathmin 1 expression induced by Epstein-Barr virus in human B lymphocytes. *Cell Proliferat* **40**, 268–281 (2007). 10.1111/j.1365-2184.2007.00429.x PMC6496458 17472732

[51] Jun, H. S. & Yoon, J. W. A new look at viruses in type 1 diabetes. *Diab. Metab. Res. Rev.* **19**, 8–31 (2003). 10.1002/dmrr.337 12592641

[52] Perez, R. K. et al. Single-cell RNA-seq reveals cell type-specific molecular and genetic associations to lupus. *Science* **376**, eabf1970 (2022). 35389781 10.1126/science.abf1970 PMC9297655

[53] Terekhova, M. et al. Single-cell atlas of healthy human blood unveils age-related loss of NKG2C(+)GZMB(-)CD8(+) memory T cells and accumulation of type 2 memory T cells. *Immunity* **56**, 2836–2854 e2839 (2023). 37963457 10.1016/j.immuni.2023.10.013

[54] Zhou, Y. et al. Metascape provides a biologist-oriented resource for the analysis of systems-level datasets. *Nat. Commun.* **10**, 1523 (2019). 30944313 10.1038/s41467-019-09234-6 PMC6447622

[55] Edgar, R., Domrachev, M. & Lash, A. E. Gene Expression Omnibus: NCBI gene expression and hybridization array data repository. *Nucleic Acids Res.* **30**, 207–210 (2002). 11752295 10.1093/nar/30.1.207 PMC99122

