# Supplementary material for: Multi-omics reveals microbiota, metabolite, and immunological heterogeneity of age-related endotypes in type 1 diabetes
Source: Signal Transduct Target Ther. 2026 Jun 15;11:233. doi: 10.1038/s41392-026-02724-2 (PMC13269897; doi:10.1038/s41392-026-02724-2)
Supplement: Supplementary file 1 — Supplementary materials for Multi-omics reveals microbiota, metabolite, and immunological heterogeneity of age-related endotypes in type 1 diabetes [file 41392_2026_2724_MOESM1_ESM.docx]

Supplementary Materials for

Multi-omics reveals microbiota, metabolite, and immunological heterogeneity of age-related endotypes in type 1 diabetes

Lanxin Pan^1#^, Huiling Tan^1#^, Tong Yue^1#,*^, Yu Ding¹, Zhaohe Gu¹, Xulin Wang¹, Jing Wang¹, Tian Wei¹, Xiaoya Zhang¹, Yu Shi¹, Shiru Chang¹, Chuang Guo^2^, Xueying Zheng^1*^, Jianping Weng^1,3*^

Correspondence to: yuetong@mail.ustc.edu.cn, lxyzheng@ustc.edu.cn, and wengjp@ustc.edu.cn

**This PDF file includes:**

Supplementary Figures. S1 to S13

Supplementary Tables S1 to S10


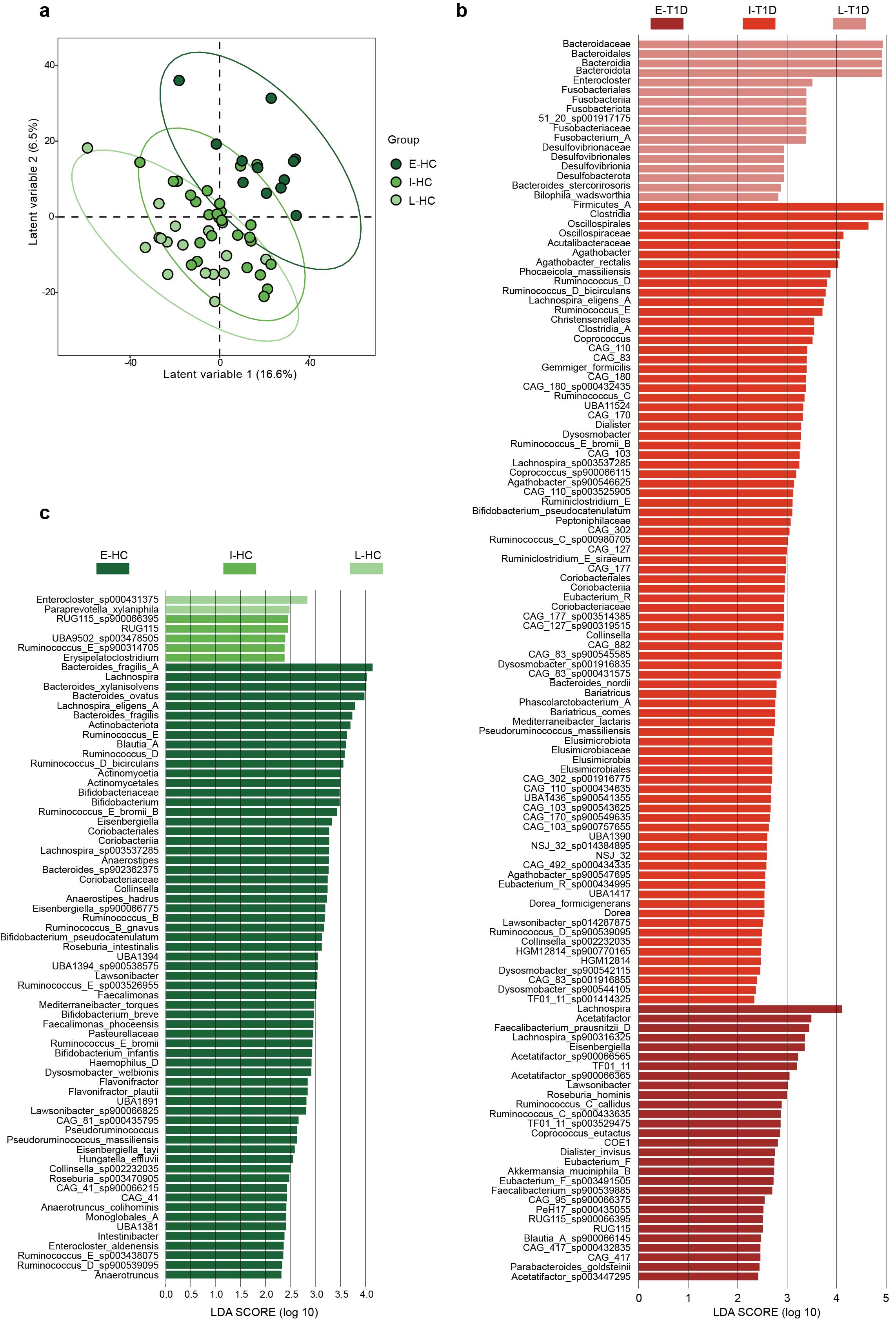


**Supplementary Figure 1. The microbial signatures in age-related endotypes of T1D**

**(a)** PLS-DA plot showing gut microbiota distribution across E-HC, I-HC, and L-HC subgroups. **(b)** LDA scores of differentially enriched taxa among E-T1D, I-T1D, and L-T1D, identified by LEfSe. **(c)** LDA scores of differentially enriched taxa among E-HC, I-HC, and L-HC, identified by LEfSe.

**
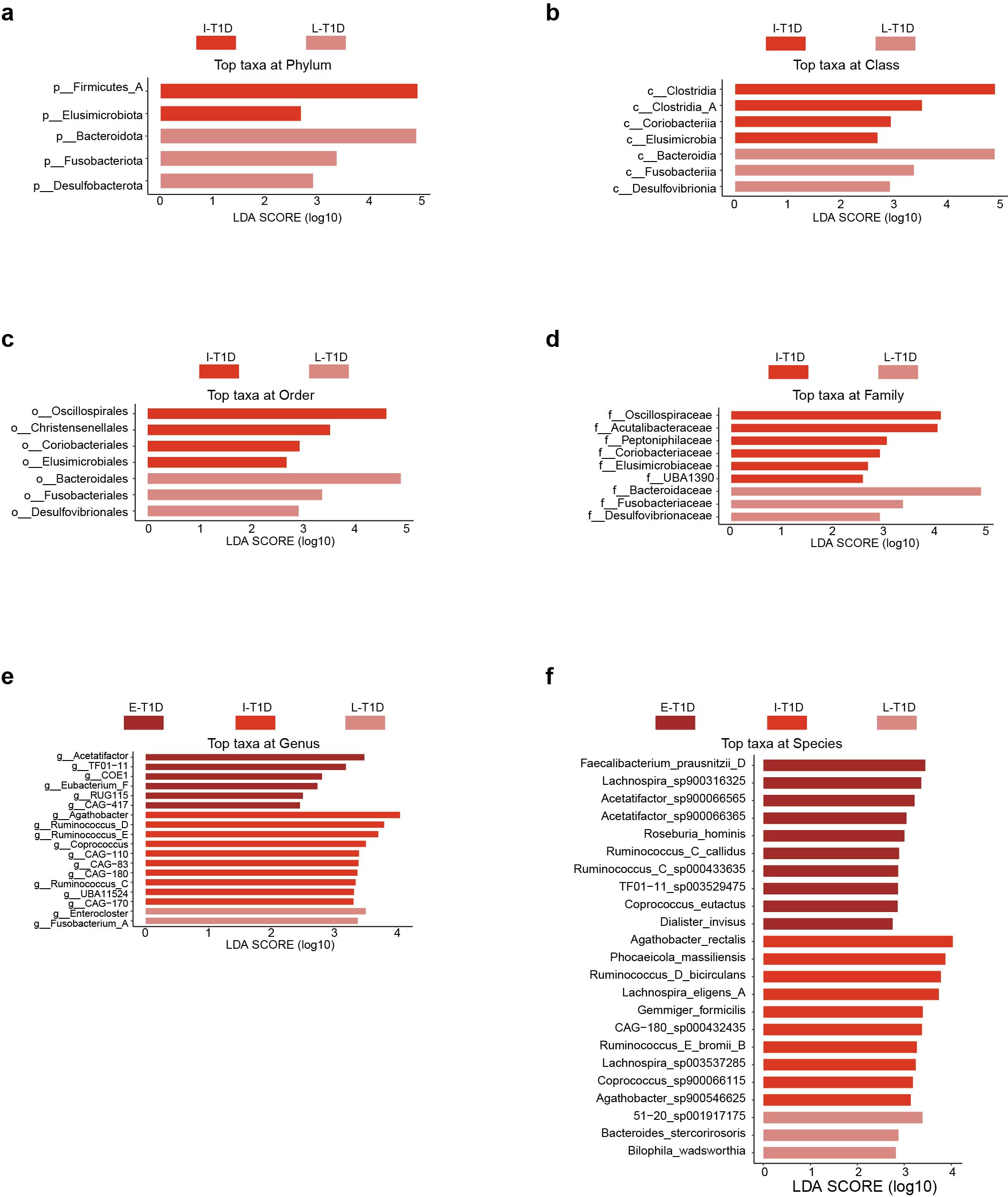
**

**Supplementary Figure 2. The microbial signatures in age-related endotypes of T1D**

Top specifically enriched microbial taxa among E-T1D, I-T1D, and L-T1D subgroups identified by LEfSe. Taxa with the highest LDA scores are shown at the phylum **(a)**, class **(b)**, order **(c)**, family **(d)**, genus **(e)**, and species **(f)** levels.

**
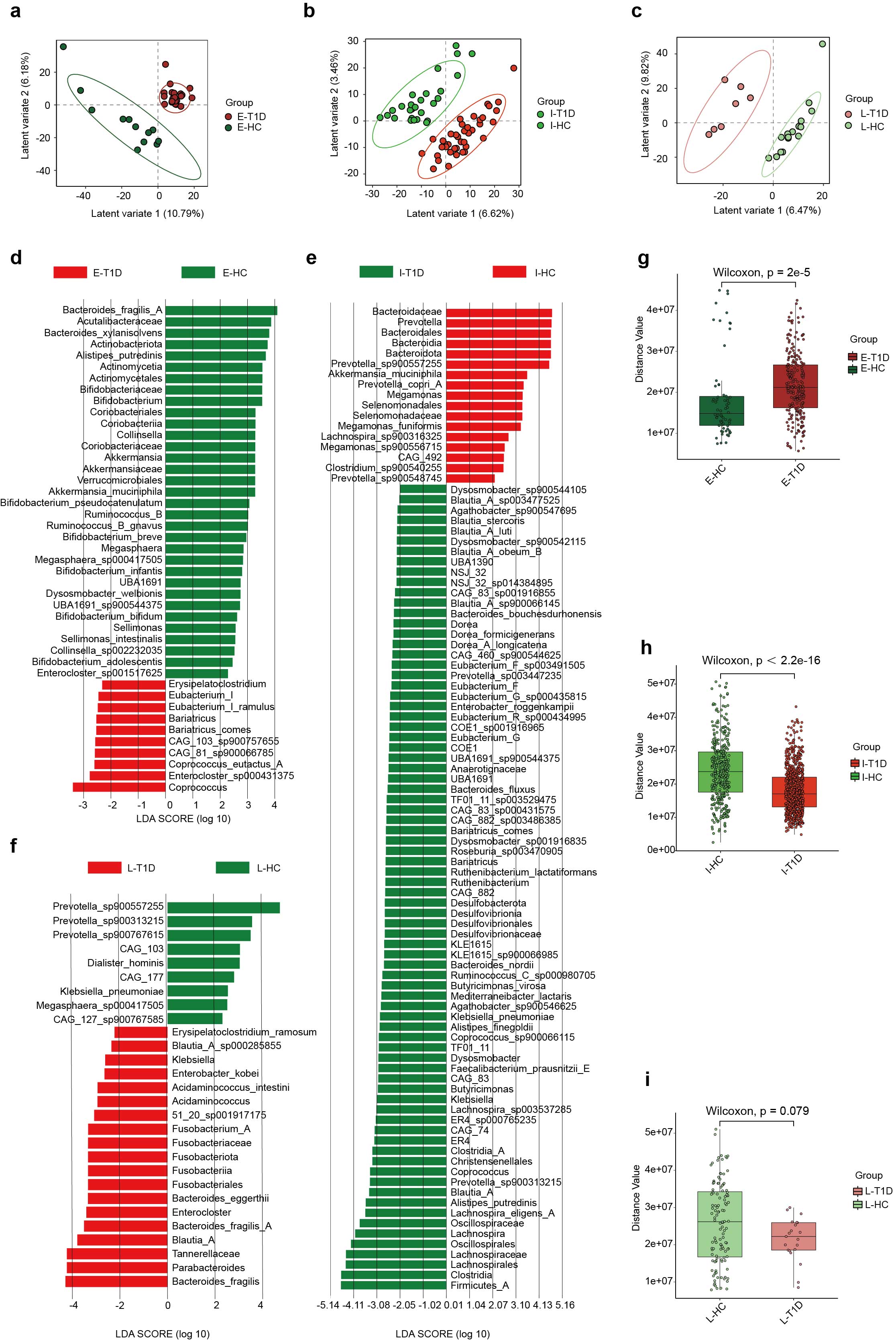
**

**Supplementary Figure 3. The microbial signatures in age-related endotypes of T1D**

(a) PLS-DA plot showing gut microbiota distribution between E-T1D and E-HC subgroups. (b) PLS-DA plot showing gut microbiota distribution between I-T1D and I-HC subgroups. (c) PLS-DA plot showing gut microbiota distribution between L-T1D and L-HC subgroups. (d) LDA scores of differentially enriched gut taxa between E-T1D and E-HC, identified by LEfSe. (e) LDA scores of differentially enriched gut taxa between I-T1D and I-HC, identified by LEfSe. (f) LDA scores of differentially enriched gut taxa between L-T1D and L-HC, identified by LEfSe. (g) β-diversity comparison between E-T1D and E-HC subgroups based on Euclidean distance. (h) β-diversity comparison between I-T1D and I-HC subgroups based on Euclidean distance. (i) β-diversity comparison between L-T1D and L-HC subgroups based on Euclidean distance. Statistical comparisons were performed using the Kruskal-Wallis test. Asterisks indicate significance levels: P < 0.05 (*), P < 0.01 (**), P < 0.001 (***), and P < 0.0001 (****); “ns” indicates not significant.


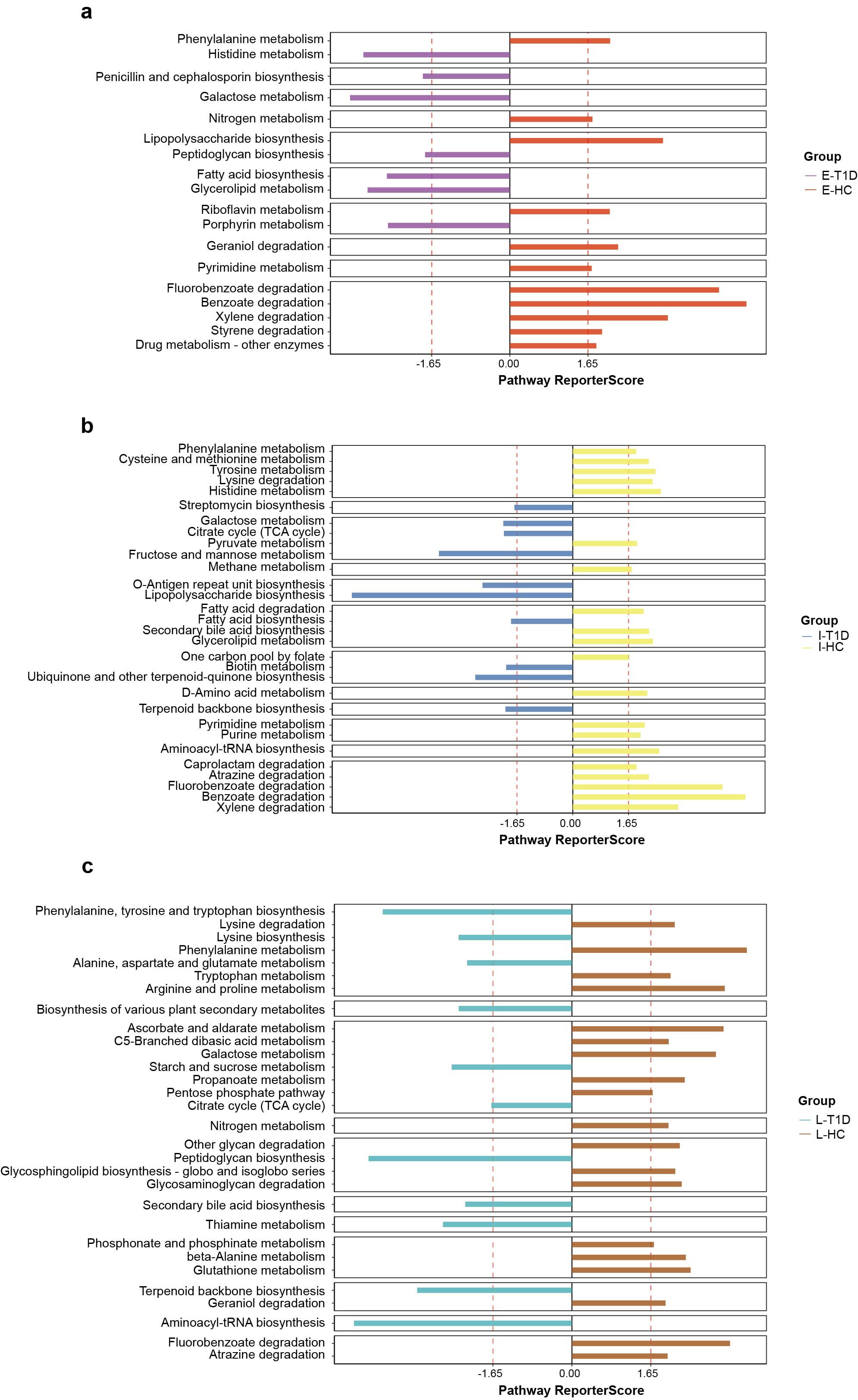


**Supplementary Figure 4. The microbial signatures in age-related endotypes of T1D**

**(a)** Pathway enrichment analysis of significantly altered gut taxa between E-T1D and E-HC subgroups. **(b)** Pathway enrichment analysis of significantly altered gut taxa between I-T1D and I-HC subgroups. **(c)** Pathway enrichment analysis of significantly altered gut taxa between L-T1D and L-HC subgroups. Colors represent different T1D or HC subgroups.


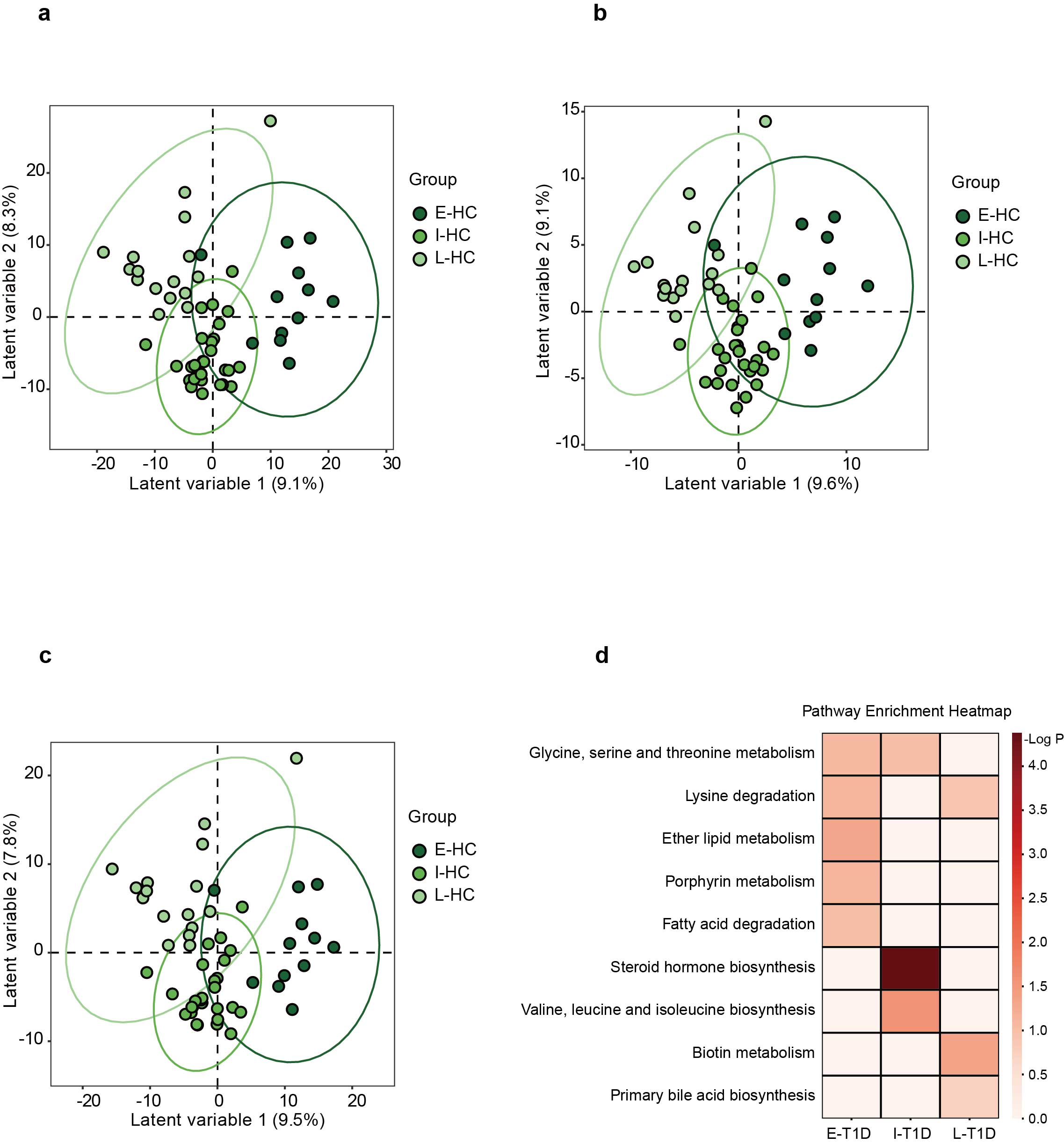


**Supplementary Figure 5. The metabolic signatures in age-related endotypes of T1D**

PLS-DA plot showing serum metabolite distribution across E-HC, I-HC, and L-HC subgroups for all identified metabolites **(a)**, endogenous metabolites **(b)** and environmental metabolites **(c)**. **(d)** Pathway enrichment analysis of subgroup-specific metabolite features among E-T1D, I-T1D, and L-T1D groups.


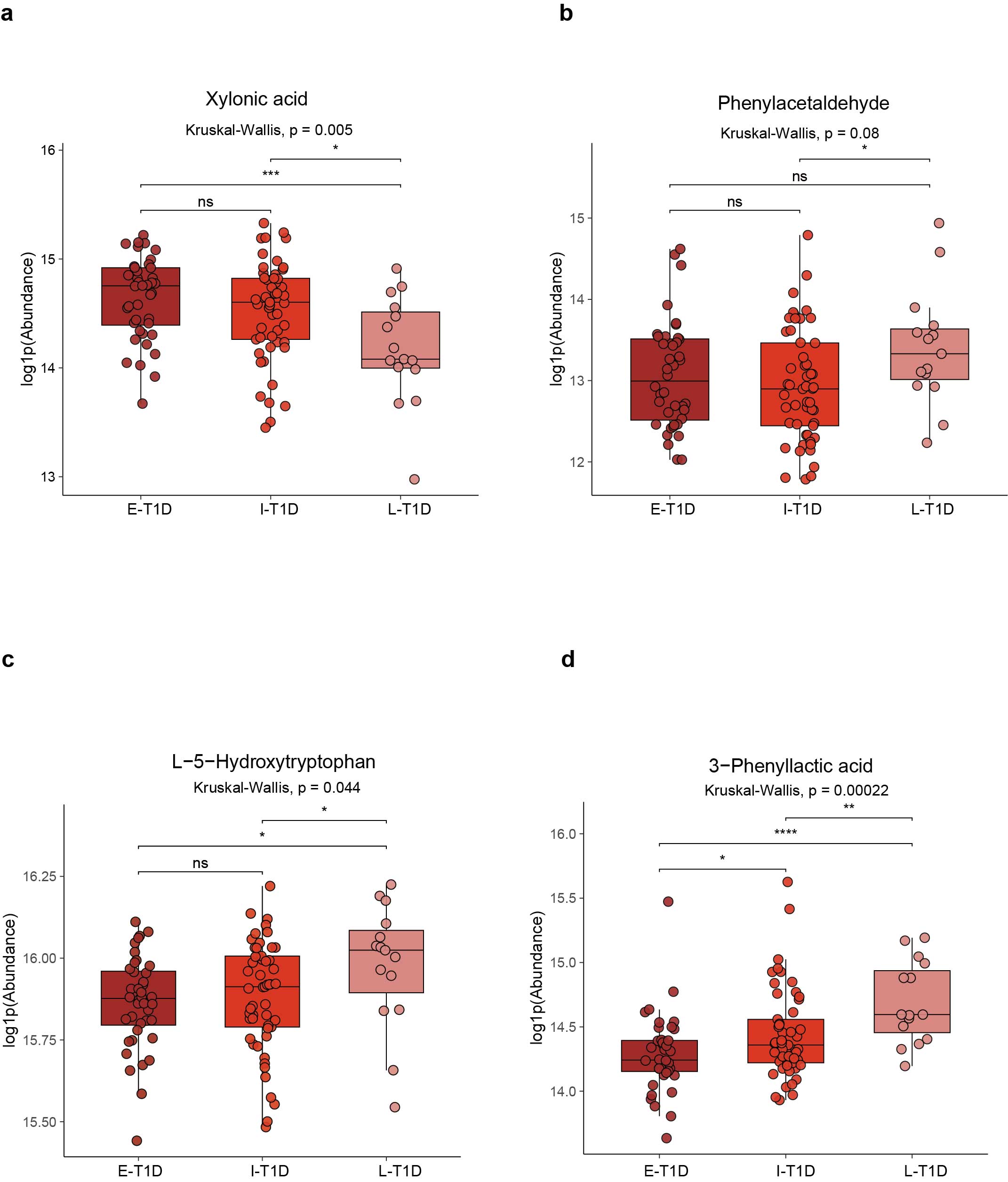


**Supplementary Figure 6. The metabolic signatures in age-related endotypes of T1D**

Comparisons of the relative abundances of four serum metabolites among the three T1D subgroups (E-T1D, I-T1D, and L-T1D). **(a)** Xylonic acid, involved in the pentose and glucuronate interconversion pathway; **(b)** Phenylacetaldehyde, **(c)** L-5-Hydroxytryptophan, and **(d)** 3-Phenyllactic acid, all associated with the phenylalanine metabolism pathway. Differences were assessed using the Kruskal-Wallis test followed by pairwise Wilcoxon tests with Holm correction for multiple comparisons.

**
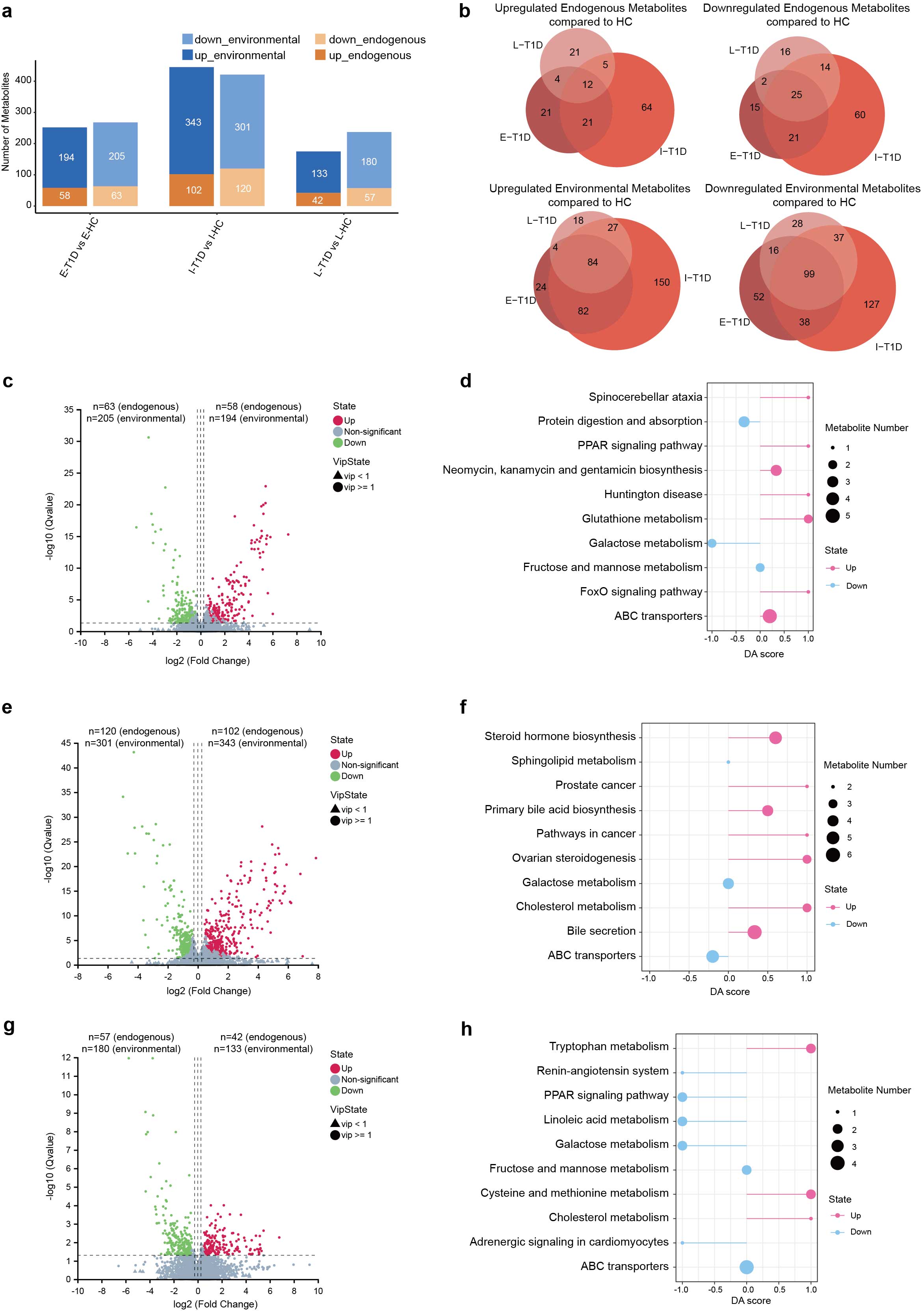
**

**Supplementary Figure 7. The metabolic signatures in age-related endotypes of T1D**

**(a)** Number of significantly altered metabolites in each pairwise group comparison between T1D and corresponding HC groups (E-T1D vs. E-HC, I-T1D vs. I-HC, L-T1D vs. L-HC), categorized by endogenous and environmental sources. **(b)** Venn diagram showing overlap of differential metabolites compared to the corresponding HC subgroup among E-T1D, I-T1D, and L-T1D, categorized by endogenous and environmental sources. **(c)** Volcano plot showing differential metabolites between E-T1D and E-HC subgroups. **(d)** Pathway enrichment analysis of significantly altered metabolites between E-T1D and E-HC subgroups. **(e)** Volcano plot showing differential metabolites between I-T1D and I-HC subgroups. **(f)** Pathway enrichment analysis of significantly altered metabolites between I-T1D and I-HC subgroups. **(g)** Volcano plot showing differential metabolites between L-T1D and L-HC subgroups. **(h)** Pathway enrichment analysis of significantly altered metabolites between L-T1D and L-HC subgroups. In panels c, e, and g, each point represents a metabolite; color indicates the direction of change (red: upregulated; grey: non-significant; green: downregulated), and shape reflects the VIP score (circle: VIP ≥ 1; triangle: VIP < 1). In panels d, f, and h, color indicates the direction of change (red: upregulated; blue: downregulated), and dot size corresponds to the number of matched metabolites.


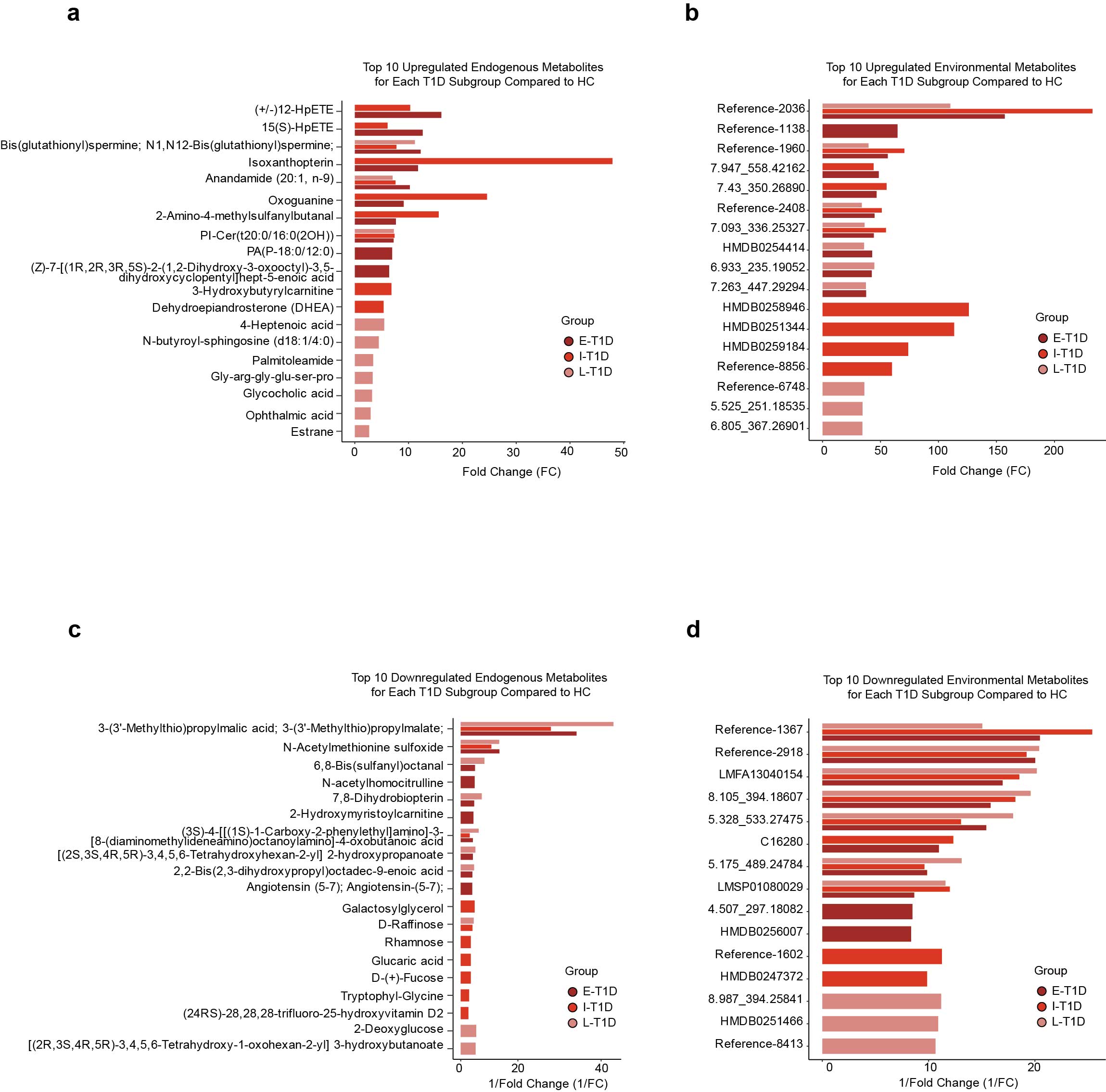


**Supplementary Figure 8. The metabolic signatures in age-related endotypes of T1D**

Top 10 differential metabolites for each T1D subgroup compared to the corresponding HC subgroup based on fold change, categorized by sources (endogenous and environmental) and directions (upregulated and downregulated).


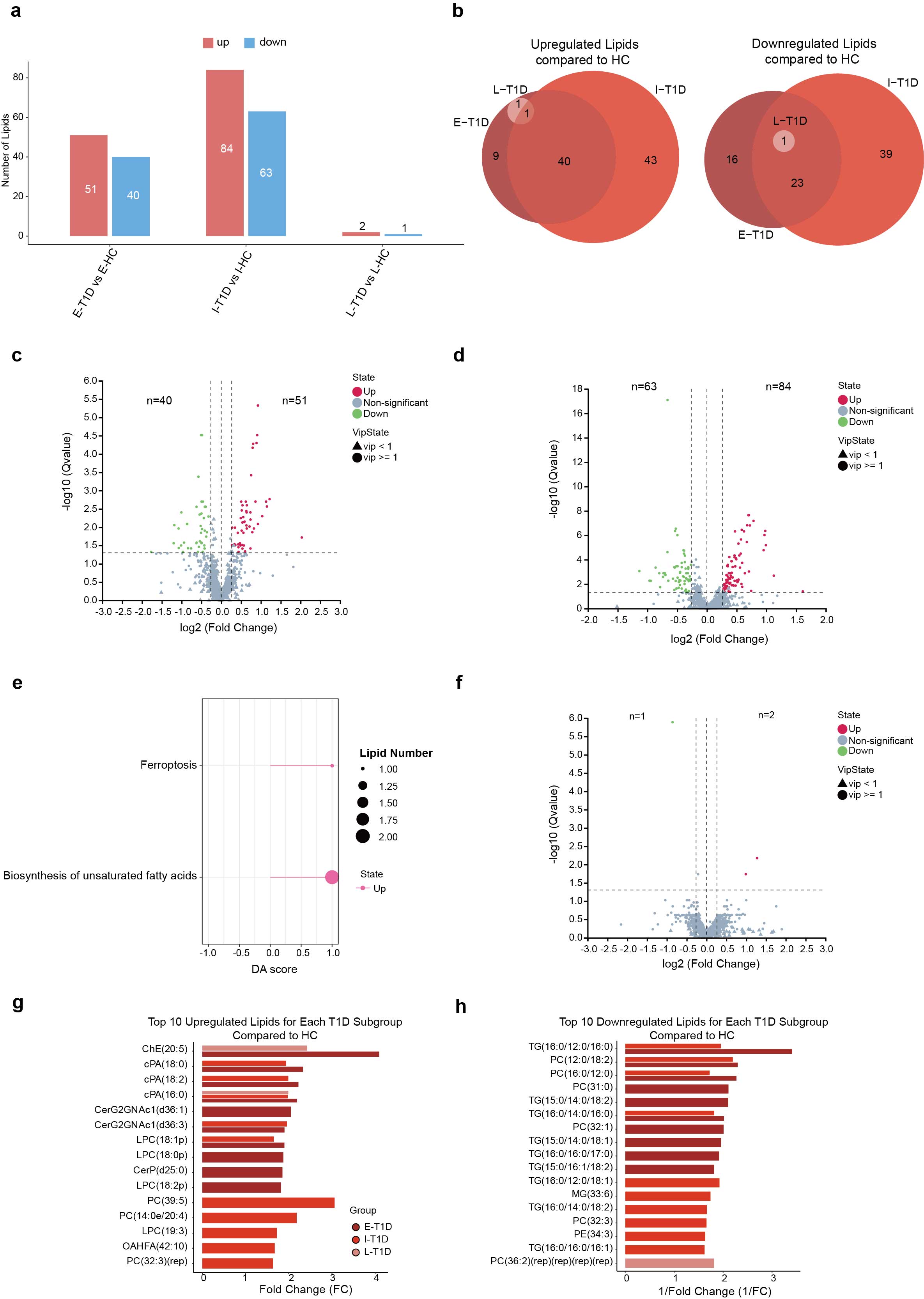


**Supplementary Figure 9. The lipidomic signatures in age-related endotypes of T1D**

**(a)** Number of significantly altered lipids in each pairwise group comparison between T1D and corresponding HC groups (E-T1D vs. E-HC, I-T1D vs. I-HC, L-T1D vs. L-HC). **(b)** Venn diagram showing overlap of differential lipids compared to the corresponding HC subgroup among E-T1D, I-T1D, and L-T1D. **(c)** Volcano plot showing differential lipids between E-T1D and E-HC subgroups. **(d)** Volcano plot showing differential lipids between I-T1D and I-HC subgroups. **(e)** Pathway enrichment analysis of significantly altered lipids between I-T1D and I-HC subgroups. **(f)** Volcano plot showing differential lipids between L-T1D and L-HC subgroups. Top 10 differential lipids for each T1D subgroup compared to the corresponding HC subgroup based on fold change, categorized by upregulation **(g)** and downregulation **(h)**. In panels c, d, and f, each point represents a lipid; color indicates the direction of change (red: upregulated; grey: non-significant; green: downregulated), and shape reflects the VIP score (circle: VIP ≥ 1; triangle: VIP < 1). In panel e, color indicates the direction of change (red: upregulated), and dot size corresponds to the number of matched lipids.


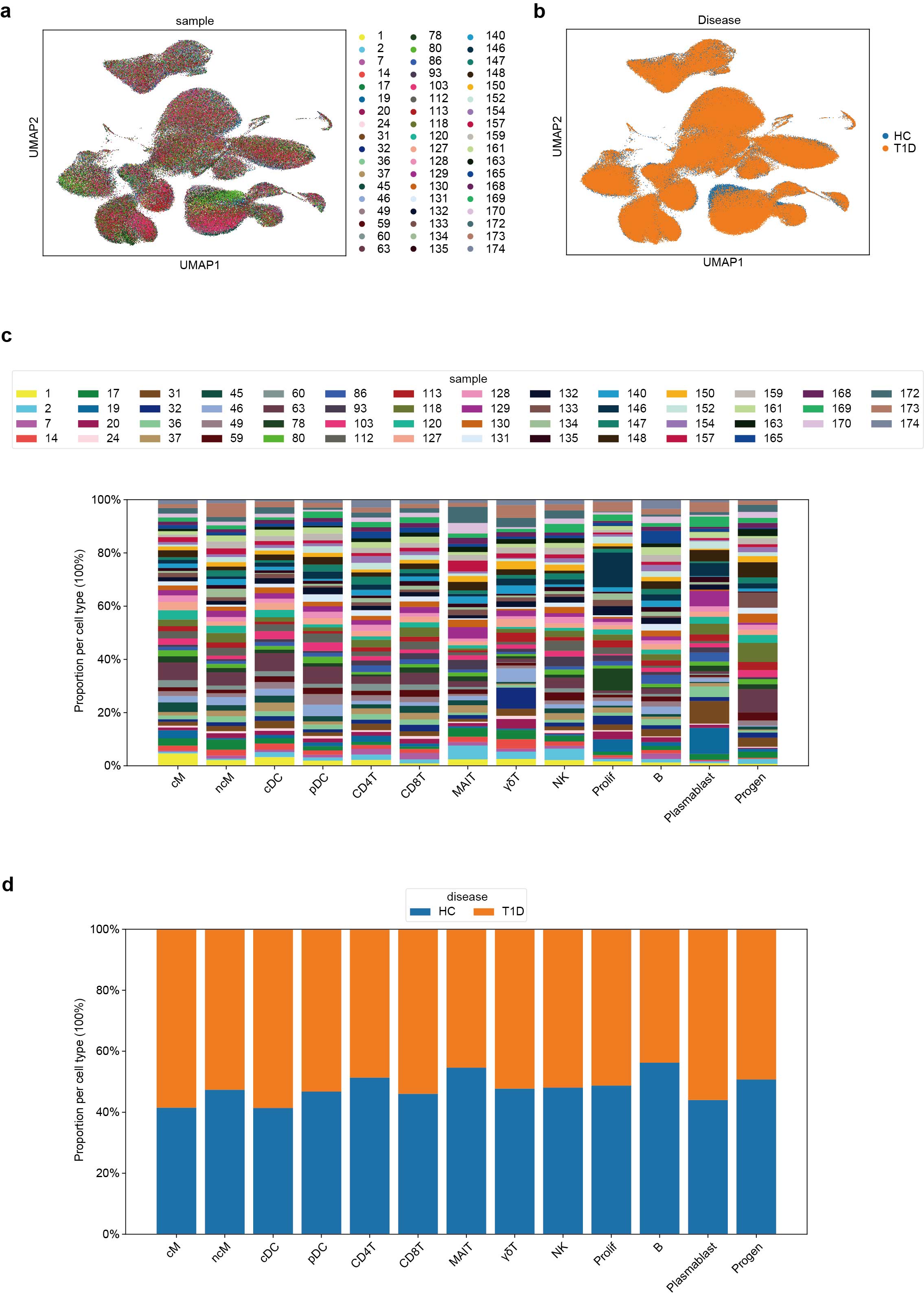


**Supplementary Figure 10. The single-cell RNA-seq signatures in age-related endotypes of T1D**

**(a)** ScRNA-seq UMAP plot of all samples colored by individual sample. **(b)** ScRNA-seq UMAP plot colored by disease status. **(c)** Stacked bar plots showing the proportion of each sample contributing to different immune cell types across all individuals. **(d)** Comparison of average immune cell type proportions between T1D and HC groups.


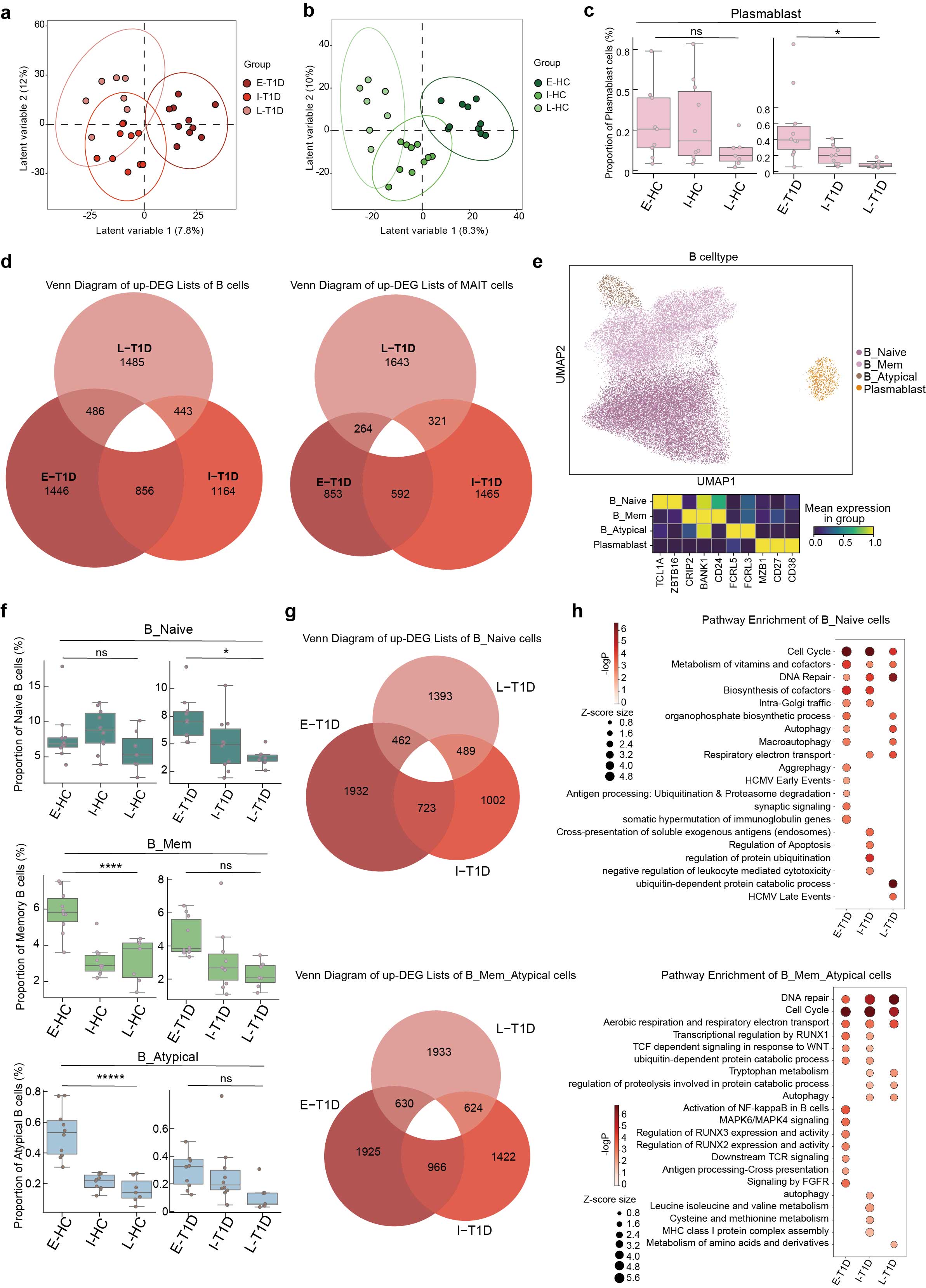


**Supplementary Figure 11. The single-cell RNA-seq signatures in age-related endotypes of T1D**

**(a)** PLS-DA plot of immune transcriptomic profiles across E-T1D, I-T1D, and L-T1D subgroups. **(b)** PLS-DA plot of immune transcriptomic profiles across E-HC, I-HC, and L-HC subgroups. **(c**) Proportions of peripheral Plasmablast cells to total lymphocytes across T1D and HC subgroups. **(e**) UMAP projection showing four B cell subtypes after reclustering of B cells, including naïve B cells (B_Naive), memory B cells (B_Mem), atypical B cells (B_Atypical), and plasmablasts (Plasmablast), with colors indicating different cell types, and a corresponding heatmap displaying representative marker gene expression across these subtypes, where colors represent scaled average expression values per group. **(f)** Proportions of peripheral B_Naive , B_Mem , and B_Atypical cells to total lymphocytes across T1D and HC subgroups. **(g)** Venn diagram of upregulated DEGs in B_Naive and B_Mem_Atypical cells across the three T1D subgroups. Colors represent different T1D subgroups. **(h)** Pathway enrichment analysis of DEGs in B_Naive and B_Mem_Atypical cells from E-T1D, I-T1D, and L-T1D groups. Colors indicate –log(P) values, and dot size indicates Z-score. Statistical analysis of cell proportion differences was performed using weighted least squares. Asterisks indicate significance levels: P < 0.05 (*), P < 0.01 (**), P < 0.001 (***), P < 0.0001 (****), and P < 0.00001 (*****); “ns” indicates not significant.


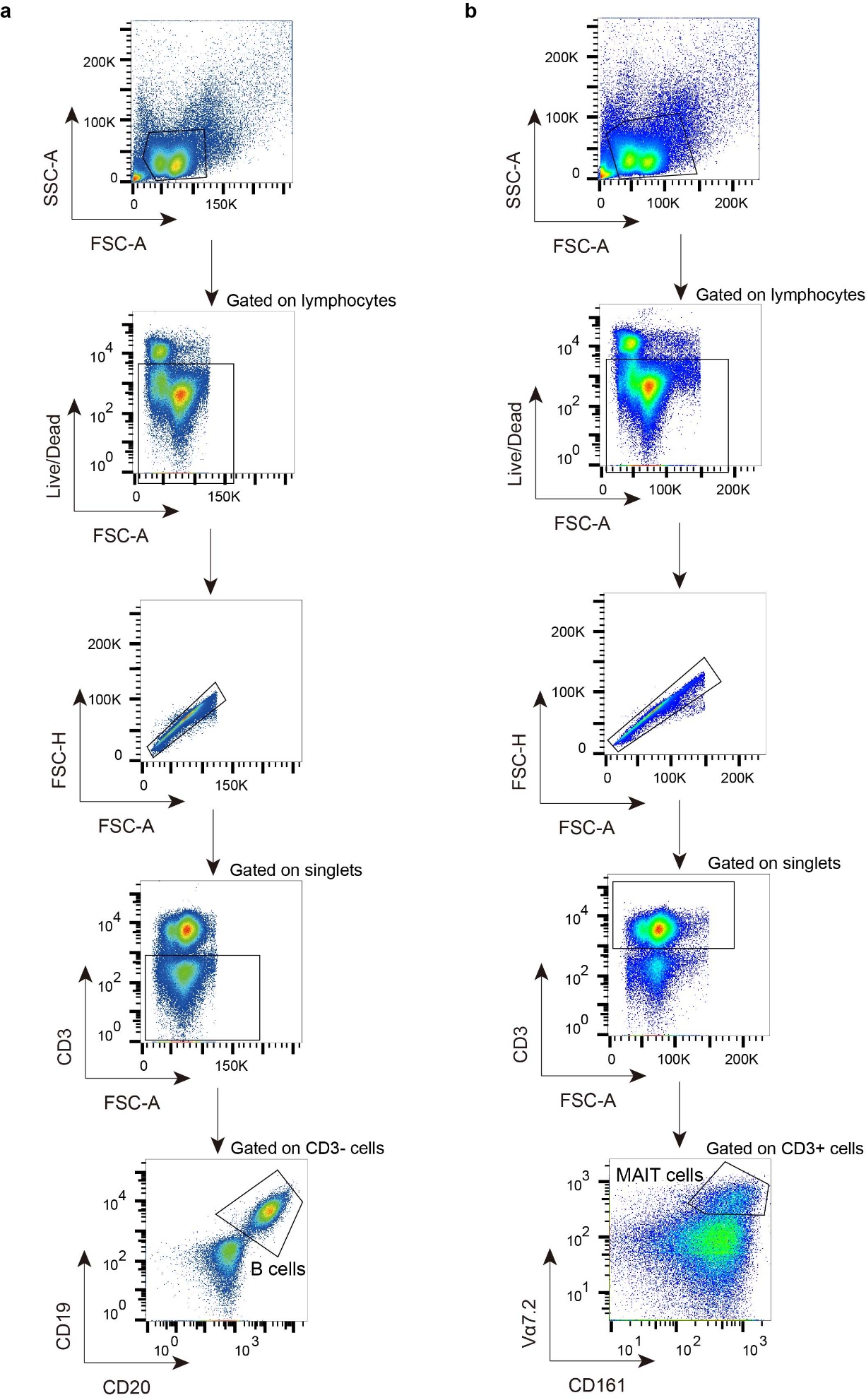


**Supplementary Figure 12. Flow cytometric gating strategy for identifying peripheral B cells and MAIT cells**

Representative flow cytometry gating strategy for **(a)** B cells and **(b)** MAIT cells. B cells were identified as CD3⁻CD19⁺CD20⁺ cells. MAIT cells were identified as CD3⁺Vα7.2⁺CD161⁺ cells.

**
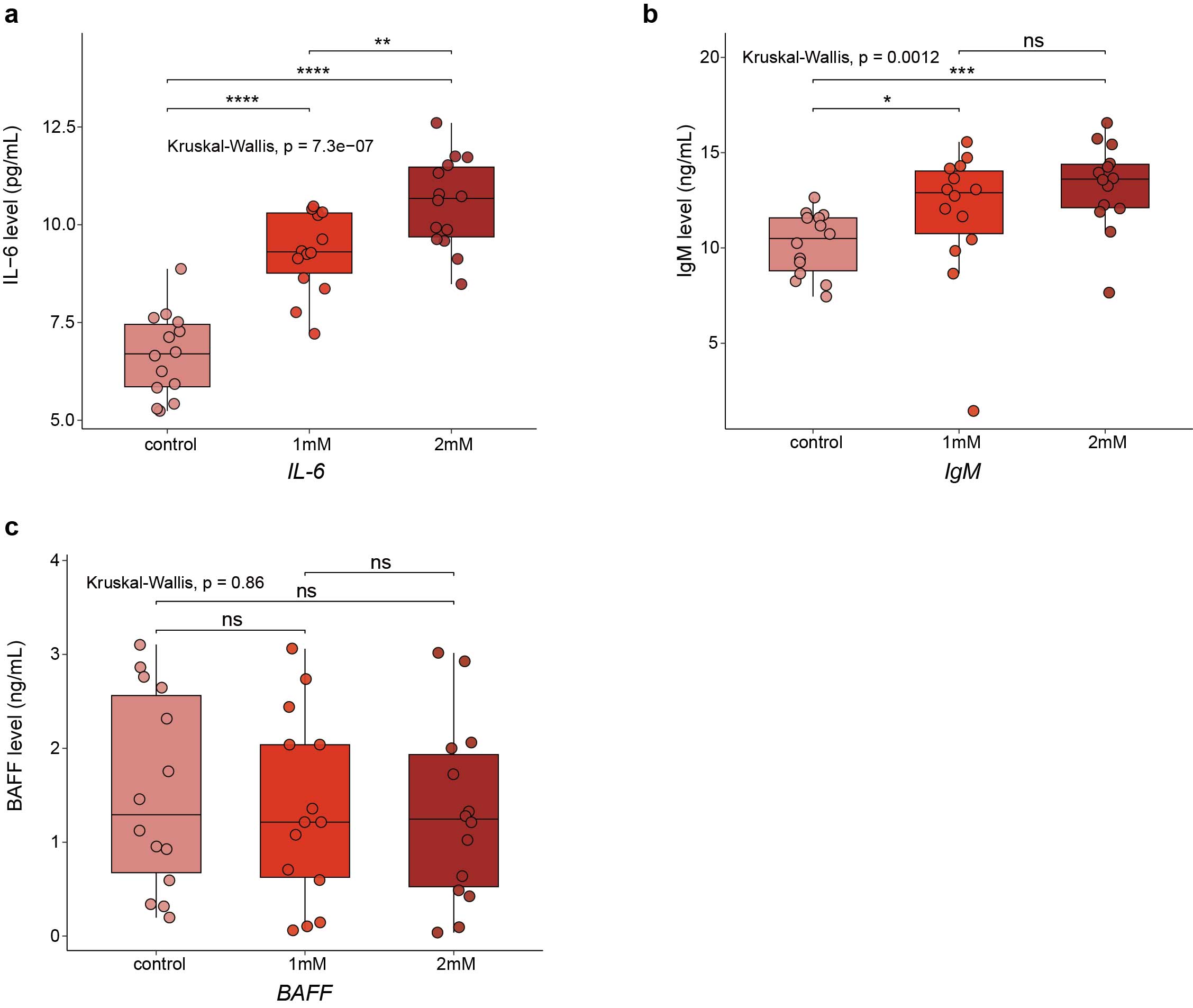
**

**Supplementary Figure 13. Effects of DPA stimulation on cytokine secretion by peripheral B cells**

Peripheral blood B cells were cultured in vitro with different concentrations of docosapentaenoic acid for 48 h, and cytokine levels in the culture supernatants were quantified by ELISA. (a) Interleukin-6 (IL-6), (b) immunoglobulin M (IgM), and (c) B-cell activating factor (BAFF) levels were compared among groups. Statistical analysis was performed using the Kruskal-Wallis test followed by pairwise Wilcoxon tests with Holm correction for multiple comparisons.

**Supplementary Tables**

| Characteristics | **E-T1D (*N=40)*** | **I-T1D (*N=53)*** | **L-T1D (*N=15)*** | ***p*.overall** |
| --- | --- | --- | --- | --- |
| Male sex | 18 (45.0%) | 27 (50.9%) | 6 (40.0%) | 0.709 |
| Age (year) | 6.70 (1.89) | 11.8 (2.05) | 16.9 (2.02) | <0.001 |
| Age at Diagnosis (year) | 4.83 (1.60) | 9.88 (1.71) | 15.2 (1.69) | <0.001 |
| Duration (year) | 1.70 [0.90;3.00] | 1.70 [1.00;2.70] | 1.50 [0.60;2.50] | 0.811 |
| Onset ketoacidosis (+) | 36 (90.0%) | 41 (77.4%) | 11 (73.3%) | 0.179 |
| HLA HR (+) | 23 (57.5%) | 18 (34.0%) | 3 (20.0%) | 0.015 |
| HLA DR3 (+) | 27 (67.5%) | 22 (41.5%) | 5 (33.3%) | 0.017 |
| HLA DR4 (+) | 17 (42.5%) | 25 (47.2%) | 7 (46.7%) | 0.899 |
| HLA DR9 (+) | 19 (47.5%) | 25 (47.2%) | 8 (53.3%) | 0.910 |
| HbA1c (%) | 6.80 [6.47;7.30] | 7.10 [6.40;8.00] | 7.10 [6.00;8.95] | 0.265 |
| GLU (mmol/L) | 7.39 [5.55;9.09] | 7.84 [5.81;10.1] | 6.08 [5.31;10.4] | 0.596 |
| ZnT8A (+) | 17 (42.5%) | 20 (37.7%) | 7 (46.7%) | 0.791 |
| IA2A (+) | 29 (72.5%) | 35 (66.0%) | 8 (53.3%) | 0.402 |
| GADA (+) | 20 (50.0%) | 36 (67.9%) | 10 (66.7%) | 0.191 |
| Fasting C-peptide (ng/mL) | 0.10 [0.10;0.19] | 0.26 [0.10;0.67] | 0.82 [0.31;1.29] | <0.001 |
| Heart rate (bpm) | 94.7 (15.3) | 90.2 (13.2) | 89.5 (18.6) | 0.284 |
| SBP (mmHg) | 96.2 (9.56) | 107 (13.4) | 115 (14.1) | <0.001 |
| DBP (mmHg) | 64.0 [59.5;66.6] | 67.8 [61.0;73.0] | 76.0 [71.3;79.0] | <0.001 |
| Weight (kg) | 21.5 [18.5;28.9] | 40.0 [30.0;48.0] | 54.0 [49.2;63.8] | <0.001 |
| Height (cm) | 120 [110;134] | 152 [142;162] | 172 [166;181] | <0.001 |
| Waistline (cm) | 53.5 [50.0;58.0] | 61.0 [56.0;68.5] | 72.0 [70.0;75.0] | <0.001 |
| Hipline (cm) | 61.6 [56.8;68.5] | 76.0 [70.0;84.5] | 90.0 [87.0;92.7] | <0.001 |
| BMI (kg/m^2^) | 15.1 [14.3;16.8] | 16.7 [15.1;18.9] | 19.8 [16.5;21.3] | <0.001 |
| WHR | 0.86 (0.05) | 0.81 (0.06) | 0.81 (0.06) | <0.001 |
| HDLC (mmol/L) | 1.71 [1.47;2.04] | 1.67 [1.39;1.95] | 1.39 [1.29;1.44] | 0.005 |
| LDLC (mmol/L) | 2.10 [1.77;2.60] | 2.22 [1.83;2.77] | 2.34 [1.75;2.48] | 0.779 |
| TG (mmol/L) | 0.55 [0.47;0.69] | 0.76 [0.60;0.98] | 0.91 [0.59;1.27] | <0.001 |
| CHOL (mmol/L) | 4.32 [3.77;4.84] | 4.14 [3.66;5.18] | 3.95 [3.28;4.44] | 0.522 |
| TPOAb | 4.92 [0.27;25.8] | 2.42 [0.25;10.7] | 9.20 [3.69;131] | 0.099 |

**Supplementary Table 1. Characteristics of the three T1D subgroups according to age at diagnosis**

Data are presented as number (%), median [interquartile range], and mean [standard deviation].

Abbreviations: T1D: type 1 diabetes; HLA: human leukocyte antigen; HR: high risk; HbA1c: haemoglobin A1c; GLU: glucose; ZnT8A: zinc transporter 8 autoantibodies; IA2A: insulinoma associated-2 autoantibodies; GADA: anti-glutamic acid decarboxylase antibodies; SBP: systolic blood pressure; DBP: diastolic blood pressure; BMI: body mass index; WHR: waist-to-hip ratio; HDLC: high-density lipoprotein cholesterol; LDLC: low-density lipoprotein cholesterol; TG: triacylglycerol; CHOL: total cholesterol; TPOAb: thyroid peroxidase antibodies.

| **Characteristics** | **E-HC (*N=12)*** | **I-HC (*N=28)*** | **L-HC (*N=16)*** | ***p*.overall** |
| --- | --- | --- | --- | --- |
| Male sex | 7 (58.3%) | 14 (50.0%) | 6 (37.5%) | 0.532 |
| Age (year) | 5.29 (1.78) | 9.69 (1.62) | 16.6 (2.57) | <0.001 |
| HbA1c (%) | 5.35 [5.18;5.50] | 5.50 [5.38;5.62] | 5.40 [5.27;5.53] | 0.140 |
| GLU (mmol/L) | 4.50 (0.50) | 4.82 (0.50) | 4.84 (0.40) | 0.123 |
| Heart rate (bpm) | 93.1 [86.4;94.6] | 89.6 [86.7;94.0] | 75.5 [68.2;78.0] | <0.001 |
| SBP (mmHg) | 108 [102;123] | 114 [104;124] | 112 [106;125] | 0.788 |
| DBP (mmHg) | 69.3 (7.83) | 69.6 (6.93) | 69.2 (8.91) | 0.990 |
| Weight (kg) | 19.9 (4.33) | 36.4 (8.47) | 56.3 (9.13) | <0.001 |
| Height (cm) | 113 (12.3) | 145 (10.2) | 172 (8.22) | <0.001 |
| Waistline (cm) | 51.6 (4.28) | 63.2 (7.04) | 70.7 (7.15) | <0.001 |
| Hipline (cm) | 61.7 (5.27) | 76.6 (6.98) | 90.0 (6.44) | <0.001 |
| BMI (kg/m^2^) | 15.5 [14.7;16.1] | 16.9 [15.6;17.9] | 18.0 [17.3;20.1] | <0.001 |
| WHR | 0.84 (0.02) | 0.83 (0.05) | 0.78 (0.05) | 0.006 |
| HDLC (mmol/L) | 1.69 (0.23) | 1.59 (0.20) | 1.68 (0.16) | 0.215 |
| LDLC (mmol/L) | 2.12 [1.90;2.34] | 1.97 [1.76;2.25] | 2.00 [1.88;2.27] | 0.379 |
| TG (mmol/L) | 0.65 [0.61;0.76] | 0.60 [0.55;0.67] | 0.68 [0.62;0.82] | 0.254 |
| CHOL (mmol/L) | 3.88 [3.85;4.72] | 3.85 [3.70;4.30] | 4.00 [3.95;4.12] | 0.315 |
| TPOAb | 0.27 [0.25;0.79] | 0.34 [0.25;1.12] | 0.49 [0.25;0.86] | 0.907 |

**Supplementary Table 2. Characteristics of the three HC subgroups according to age**

Data are presented as number (%), median [interquartile range], and mean [standard deviation].

Abbreviations: HC: healthy control; HbA1c: haemoglobin A1c; GLU: glucose; SBP: systolic blood pressure; DBP: diastolic blood pressure; BMI: body mass index; WHR: waist-to-hip ratio; HDLC: high-density lipoprotein cholesterol; LDLC: low-density lipoprotein cholesterol; TG: triacylglycerol; CHOL: total cholesterol; TPOAb: thyroid peroxidase antibodies.

| **Characteristics** | **E-HC (*N=12)*** | **E-T1D**  **(N=40)** | **P** | **I-HC (*N=28)*** | **I-T1D**  **(N=53)** | **P** | **L-HC (N=16)** | **L-T1D (N=15)** | **P** |
| --- | --- | --- | --- | --- | --- | --- | --- | --- | --- |
| Male sex | 7 (58.3%) | 18 (45.0%) | 0.418 | 14 (50.0%) | 27 (50.9%) | 0.936 | 6 (37.5%) | 6 (40.0%) | 0.886 |
| Age (year) | 6.15 [4.65;6.53] | 6.45 [5.47;8.10] | 0.073 | 10.00 [7.97;10.93] | 11.70 [10.40;13.10] | <0.001 | 16.05 [14.78;18.62] | 16.70 [15.55;18.35] | 0.663 |
| HLA HR (+) | 2 (16.7%) | 23 (57.5%) | 0.0130 | 0 (0.0%) | 18 (34.0%) | <0.001 | 0 (0.0%) | 3 (20.0%) | 0.101 |
| HLA DR3 (+) | 2 (16.7%) | 27 (67.5%) | 0.002 | 2 (7.1%) | 22 (41.5%) | 0.001 | 3 (18.8%) | 5 (33.3%) | 0.433 |
| HLA DR4 (+) | 4 (33.3%) | 17 (42.5%) | 0.741 | 2 (7.1%) | 25 (47.2%) | <0.001 | 6 (37.5%) | 7 (46.7%) | 0.605 |
| HLA DR9 (+) | 2 (16.7%) | 19 (47.5%) | 0.093 | 8 (28.6%) | 25 (47.2%) | 0.105 | 7 (43.8%) | 8 (53.3%) | 0.594 |
| HbA1c (%) | 5.35 [5.18;5.50] | 6.80 [6.47;7.30] | <0.001 | 5.50 [5.38;5.62] | 7.10 [6.40;8.00] | <0.001 | 5.40 [5.27;5.53] | 7.10 [6.00;8.95] | <0.001 |
| GLU (mmol/L) | 4.61 [4.44;4.81] | 7.39 [5.55;9.09] | <0.001 | 4.82 [4.42;5.10] | 7.84 [5.81;10.11] | <0.001 | 4.86 [4.49;5.18] | 6.08 [5.31;10.39] | 0.001 |
| Heart rate (bpm) | 93.12 [86.40;94.56] | 94.22 [86.48;102.25] | 0.318 | 89.59 [86.65;94.00] | 90.00 [82.00;96.00] | 0.929 | 75.50 [68.25;78.00] | 83.00 [77.94;97.56] | 0.009 |
| SBP (mmHg) | 111.49 (13.42) | 96.20 (9.56) | 0.002 | 113.94 (12.41) | 106.79 (13.38) | 0.020 | 115.38 (18.35) | 115.42 (14.07) | 0.994 |
| DBP (mmHg) | 70.50 [62.65; 75.13] | 64.00 [59.50; 66.59] | 0.040 | 72.52 [63.00; 73.77] | 67.80 [61.00; 73.00] | 0.283 | 70.50 [64.75; 74.25] | 76.00 [71.27; 79.00] | 0.082 |
| Weight (kg) | 21.15 [16.70;22.13] | 21.50 [18.50;28.85] | 0.139 | 34.60 [29.77;41.00] | 40.00 [30.00;48.00] | 0.166 | 55.10 [50.67;63.97] | 54.00 [49.20;63.75] | 0.953 |
| Height (cm) | 117.50 [107.88;122.00] | 120.50 [110.00;133.50] | 0.108 | 144.00 [137.75;155.00] | 152.50 [142.00;162.50] | 0.015 | 171.75 [169.62;177.88] | 171.50 [166.00;180.75] | 0.874 |
| Waistline (cm) | 51.50 [49.50;54.25] | 53.50 [50.00;58.00] | 0.263 | 61.50 [58.30;66.12] | 61.00 [56.00;68.50] | 0.502 | 69.90 [67.25;74.70] | 72.00 [70.00;75.00] | 0.235 |
| Hipline (cm) | 61.00 [58.75;66.03] | 61.65 [56.75;68.50] | 0.853 | 77.45 [71.00;80.25] | 76.00 [70.00;84.50] | 0.925 | 90.15 [87.12;93.25] | 90.00 [87.00;92.65] | 0.828 |
| BMI (kg/m^2^) | 15.55 [14.73;16.07] | 15.07 [14.25;16.80] | 0.786 | 16.87 [15.62;17.90] | 16.72 [15.09;18.90] | 0.925 | 17.97 [17.26;20.11] | 19.79 [16.47;21.32] | 0.921 |
| WHR | 0.84 [0.83;0.85] | 0.86 [0.84;0.89] | 0.032 | 0.81 [0.79;0.86] | 0.81 [0.78;0.84] | 0.340 | 0.77 [0.76;0.81] | 0.80 [0.78;0.83] | 0.144 |
| HDLC (mmol/L) | 1.65 [1.53;1.81] | 1.71 [1.47;2.04] | 0.625 | 1.55 [1.50;1.71] | 1.67 [1.39;1.95] | 0.416 | 1.70 [1.61;1.81] | 1.39 [1.29;1.44] | <0.001 |
| LDLC (mmol/L) | 2.12 [1.90;2.34] | 2.10 [1.77;2.60] | 0.922 | 1.97 [1.76;2.25] | 2.22 [1.83;2.77] | 0.023 | 2.00 [1.88;2.27] | 2.34 [1.75;2.48] | 0.220 |
| TG (mmol/L) | 0.65 [0.61;0.76] | 0.55 [0.47;0.69] | 0.065 | 0.60 [0.55;0.67] | 0.76 [0.60;0.98] | 0.007 | 0.68 [0.62;0.82] | 0.91 [0.59;1.27] | 0.199 |
| CHOL (mmol/L) | 3.88 [3.85;4.72] | 4.32 [3.77;4.84] | 0.888 | 3.85 [3.70;4.30] | 4.14 [3.66;5.18] | 0.119 | 4.00 [3.95;4.12] | 3.95 [3.27;4.44] | 0.707 |
| TPOAb | 0.27 [0.25;0.79] | 4.92 [0.27;25.83] | 0.011 | 0.34 [0.25;1.12] | 2.42 [0.25;10.72] | 0.048 | 0.49 [0.25;0.86] | 9.20 [3.69;131.50] | <0.001 |

**Supplementary Table 3. Characteristics of T1D subgroups and their corresponding age-matched HC controls**

Data are presented as number (%), median [interquartile range], and mean [standard deviation].

Abbreviations: HC: healthy control; T1D: type 1 diabetes; HLA: human leukocyte antigen; HR: high risk; HbA1c: haemoglobin A1c; GLU: glucose; SBP: systolic blood pressure; DBP: diastolic blood pressure; BMI: body mass index; WHR: waist-to-hip ratio; HDLC: high-density lipoprotein cholesterol; LDLC: low-density lipoprotein cholesterol; TG: triacylglycerol; CHOL: total cholesterol; TPOAb: thyroid peroxidase antibodies.

| Characteristics | **E-T1D (*N=10)*** | **I-T1D (*N=10)*** | **L-T1D (*N=7)*** | ***p*.overall** |
| --- | --- | --- | --- | --- |
| Male sex | 5 (50.0%) | 5 (50.0%) | 2 (28.6%) | 0.617 |
| Age (year) | 5.8 (1.20) | 10.6 (0.84) | 17.5 (1.61) | <0.001 |
| Age at Diagnosis (year) | 5.15 (1.28) | 10.2 (0.86) | 14.5 (1.77) | <0.001 |
| Duration (year) | 0.8 [0.43;0.90] | 0.6 [0.35;1.10] | 2.3 [0.60;2.50] | 0.185 |
| Onset ketoacidosis (+) | 7 (70.0%) | 6 (60.0%) | 5 (71.4%) | 0.852 |
| HLA HR (+) | 5 (50.0%) | 6 (60.0%) | 2 (28.6%) | 0.438 |
| HLA DR3 (+) | 6 (60.0%) | 6 (60.0%) | 2 (28.6%) | 0.359 |
| HLA DR4 (+) | 3 (30.0%) | 7 (70.0%) | 5 (71.4%) | 0.122 |
| HLA DR9 (+) | 6 (60.0%) | 3 (30.0%) | 3 (42.9%) | 0.400 |
| HbA1c (%) | 7.05 [6.82;7.82] | 7.35 [7.00;8.25] | 7.10 [5.75;8.15] | 0.75 |
| GLU (mmol/L) | 7.04 [5.78;7.93] | 7.72 [4.93;8.35] | 6.08 [5.20;10.40] | 0.855 |
| ZnT8A (+) | 6 (60.0%) | 5 (50.0%) | 3 (42.9%) | 0.776 |
| IA2A (+) | 8 (80.0%) | 8 (80.0%) | 4 (57.1%) | 0.494 |
| GADA (+) | 6 (60.0%) | 9 (90.0%) | 5 (71.4%) | 0.305 |
| Heart rate (bpm) | 91.4 (24.7) | 90.2 (14.1) | 81.8 (11.5) | 0.617 |
| SBP (mmHg) | 94.6 (7.48) | 101 (13.6) | 113 (18.3) | 0.018 |
| DBP (mmHg) | 62.0 [57.2;63.7] | 64.0 [58.5;70.3] | 76.0 [71.3;77.2] | 0.041 |
| Weight (cm) | 20.5 [18.9;22.9] | 35.3 [29.1;39.9] | 58.2 [48.7;63.8] | <0.001 |
| Height (cm) | 119 [117;122] | 144 [141;149] | 175 [168;180] | <0.001 |
| Waistline (cm) | 54.5 [53.0;55.8] | 59.0 [56.2;65.8] | 73.0 [68.5;75.0] | <0.001 |
| Hipline (cm) | 60.2 [60.0;62.0] | 70.0 [70.0;78.2] | 90.0 [88.8;91.5] | <0.001 |
| BMI | 15.1 [14.6;16.1] | 16.3 [15.1;17.8] | 19.8 [16.4;21.0] | 0.071 |
| WHR | 0.89 (0.03) | 0.82 (0.07) | 0.80 (0.08) | 0.018 |
| HDLC (mmol/L) | 1.68 [1.34;1.78] | 1.74 [1.57;1.91] | 1.44 [1.27;1.44] | 0.093 |
| LDLC (mmol/L) | 2.33 [2.20;2.45] | 2.42 [2.06;3.02] | 1.84 [1.54;2.32] | 0.216 |
| TG (mmol/L) | 0.67 [0.49;0.90] | 0.64 [0.60;0.70] | 1.11 [0.71;1.27] | 0.170 |
| CHOL (mmol/L) | 4.13 [3.88;4.37] | 4.14 [3.85;5.53] | 3.27 [2.89;4.14] | 0.113 |
| TPOAb | 1.38 [0.25;8.46] | 5.02 [1.14;8.69] | 11.0 [7.23;351] | 0.230 |

**Supplementary Table 4 Characteristics of the three T1D subgroups according to age at diagnosis in the analysis of scRNA-seq**

Data are presented as number (%), median [interquartile range], and mean [standard deviation].

Abbreviations: T1D: type 1 diabetes; HLA: human leukocyte antigen; HR: high risk; HbA1c: haemoglobin A1c; GLU: glucose; ZnT8A: zinc transporter 8 autoantibodies; IA2A: insulinoma associated-2 autoantibodies; GADA: anti-glutamic acid decarboxylase antibodies; SBP: systolic blood pressure; DBP: diastolic blood pressure; BMI: body mass index; WHR: waist-to-hip ratio; HDLC: high-density lipoprotein cholesterol; LDLC: low-density lipoprotein cholesterol; TG: triacylglycerol; CHOL: total cholesterol; TPOAb: thyroid peroxidase antibodies.

| **Characteristics** | **E-HC (*N=10)*** | **I-HC (*N=10)*** | **L-HC (*N=7)*** | ***p*.overall** |
| --- | --- | --- | --- | --- |
| Male sex | 5 (50.0%) | 5 (50.0%) | 2 (28.6%) | 0.617 |
| Age (year) | 6.15 (1.60) | 10.8 (0.39) | 17.9 (2.37) | <0.001 |
| HbA1c (%) | 5.35 [5.20;5.48] | 5.50 [5.40;5.60] | 5.40 [5.25;5.50] | 0.263 |
| GLU (mmol/L) | 4.61 (0.21) | 4.89 (0.45) | 5.10 (0.49) | 0.214 |
| Heart rate (bpm) | 93.1 [82.4;94.0] | 89.5 [88.0;90.8] | 76.0 [70.5;79.5] | 0.016 |
| SBP (mmHg) | 108 [103;121] | 115 [98;127] | 112 [106;119] | 0.903 |
| DBP (mmHg) | 70.5 (8.04) | 64.5 (8.44) | 71.0 (8.45) | 0.925 |
| Weight (cm) | 21.2 (4.25) | 40.0 (4.61) | 57.8 (9.35) | <0.001 |
| Height (cm) | 118 (12.1) | 151 (5.71) | 174 (6.19) | <0.001 |
| Waistline (cm) | 51.5 (4.40) | 61.5 (3.19) | 72.0 (4.66) | <0.001 |
| Hipline (cm) | 61.0 (5.26) | 79.5 (4.38) | 90.8 (5.41) | <0.001 |
| BMI | 15.5 [14.4;16.1] | 17.3 [16.3;18.1] | 18.0 [17.4;21.0] | 0.002 |
| WHR | 0.84 (0.02) | 0.80 (0.04) | 0.78 (0.05) | 0.011 |
| HDLC (mmol/L) | 1.65 (0.24) | 1.63 (0.30) | 1.68 (0.15) | 0.935 |
| LDLC (mmol/L) | 2.12 [1.94;2.26] | 2.04 [1.86;2.29] | 2.02 [1.97;2.32] | 0.619 |
| TG (mmol/L) | 0.65 [0.61;0.72] | 0.60 [0.57;1.05] | 0.67 [0.57;0.75] | 0.960 |
| CHOL (mmol/L) | 3.88 [3.86;4.60] | 4.02 [3.82;4.40] | 4.01 [3.96;4.30] | 0.839 |
| TPOAb | 0.37 [0.25;1.23] | 0.40 [0.27;1.27] | 0.85 [0.25;1.91] | 0.966 |

**Supplementary Table 5 Characteristics of the three HC subgroups according to age at diagnosis in the analysis of scRNA-seq**

Data are presented as number (%), median [interquartile range], and mean [standard deviation].

Abbreviations: HC: healthy control; HbA1c: haemoglobin A1c; GLU: glucose; SBP: systolic blood pressure; DBP: diastolic blood pressure; BMI: body mass index; WHR: waist-to-hip ratio; HDLC: high-density lipoprotein cholesterol; LDLC: low-density lipoprotein cholesterol; TG: triacylglycerol; CHOL: total cholesterol; TPOAb: thyroid peroxidase antibodies.

**Supplementary Table 6**

Subgroup-specific and shared upregulated biomarkers in the microbiota, metabolome, lipidome, and B-cell transcriptome across T1D subgroups involved in multi-omics networks

**Supplementary Table 7**

Correlation analysis results of microbiota-metabolite/lipid interaction pairs, metabolite/lipid-gene interaction pairs, and microbiota-gene interaction pairs

**Supplementary Table 8**

Correlation results between connected microbiota, metabolites, lipids, and genes in the multi-omics networks and clinical variables

**Supplementary Table 9**

Metabolites and lipids IDs with corresponding names

**Supplementary Table 10**

Sample sizes for multi-omics analyses and detailed sample information for experimental assays
